# Supplementary material for: Commonalities and differences in injured patient experiences of accessing and receiving quality injury care: a qualitative study in three sub-Saharan African countries
Source: BMJ Open. 2024 Jul 1;14(7):e082098. doi: 10.1136/bmjopen-2023-082098 (PMC11218010; doi:10.1136/bmjopen-2023-082098)
Supplement: Supplementary data [file bmjopen-2023-082098supp001.pdf]

Appendices

Appendix 1. The English version of the topic guide

Equitable access to quality trauma systems in Lower and Middle Income Countries. Assessing gaps and developing priorities

Service User In-Depth Interview Topic Guide Version 1. 25-01-2020 (note this is a discussion guide, the research assistants running the study will be fluent in English and local languages, and will translate discussion points, where needed, for participants)

First, I would like to thank you for attending this interview.

The recordings will be anonymous, and we don't want you to say your name at the start, but we have given each of you a pseudonym (made up name), and will go around the table in turn asking you to say your pseudonym, what age you are, the area in which you live.

This interview is to understand what would happen in your community if someone suffered an injury.

I would like to think particularly about four different aspects of what would happen, these aspects are seeking care (the time from an injury happens to taking action to get care), reaching care (the time until a facility or hospital that can treat the injury is reached), receiving quality care (getting good quality treatment for the injury at a facility or hospital), and remaining in care (for example, attending follow up at out-patients or going to rehabilitation).

I wish to know about your experiences and also your thoughts on whether others in your community have similar experiences.

I realise that you have had an injury in the last 6 months, could you tell us about what happened to cause the injury?

Where were you when the injury happened?

How about after your injury happened, what did you do?
Prompts, if needed:
- Did you think that you needed help?
- Was anyone there to help?
- Were you able to telephone for help?

Did you think that you needed to go and get treatment?
(If not, why not?)

Were there any things that stopped you trying to get treatment?
Prompts, if needed
- Knowledge of availability of healthcare?
- Trust in healthcare?
- Fear of costs?
- Availability of a phone?
- No time to go to healthcare?

Where did you first go to for treatment?
Prompts, if needed:
- Is that a clinic or a hospital?
- How far away was it?
- How long did it take to get there?
- If it took a long time to reach the care, why was that?

Was that the only place you went to, or did you need to (or were you referred to) another place to get care?

Note to interviewer: please explore how many places the person needed to go to in order to get definitive care

Were there any things in particular that delayed you getting to a hospital or clinic for care?
Prompts, if needed:
- Ambulance availability?
- Road conditions?
- Personal safety?
- Costs?

How about the care that you got in the facilities that you attended – do you think that the medical treatment was good?
Prompts, if needed:
- Were you seen quickly?
- Did the doctors and nurses seem competent?
- Do you think that they had all that they needed to treat your injury well?

How about how they treated you?
Prompts, if needed:
- Were you treated with respect?
- Did the staff explain things to you?

How about costs of care?
Prompts, if needed:
- Did you have to pay for care?
- Did you have to borrow or sell anything to pay for care?

Were there any things in particular that stopped you getting the right treatment in the hospital?
Prompts if needed:
- Family commitments meant had to shorten stay?
- Costs of care?
- Too far for relatives to travel to visit?

After your injury was treated, were you asked to go back to see healthcare workers about your injury? For example, doctors in a clinical appointment, or physiotherapists for rehabilitation?

If so, how far did you have to travel to get to these appointments?

Did you manage to keep all of the appointments?

Were there any things in particular that you felt limited your ability to keep all these appointments?
Prompts, if needed:
- Travel time?
- Costs?
- Other commitments?
- Thought there was no need?

Are you now back to your full fitness, do you think?

Do you think anything could have been done better?

Now that's the end of this discussion, thank-you for telling us about your experiences.
Now, we would be grateful if we could ask you some other questions about your time in the hospital or clinic. It will take another ten minutes.

Note to interviewer ask I-PAHC and/or O-PAHC questionnaire depending on whether the participant was admitted to hospital or was seen in a clinic, or both.

Does you have any questions for me?
Thank-you

Appendix 2. Barriers to good patient experience of trauma care, in descending order of frequency.

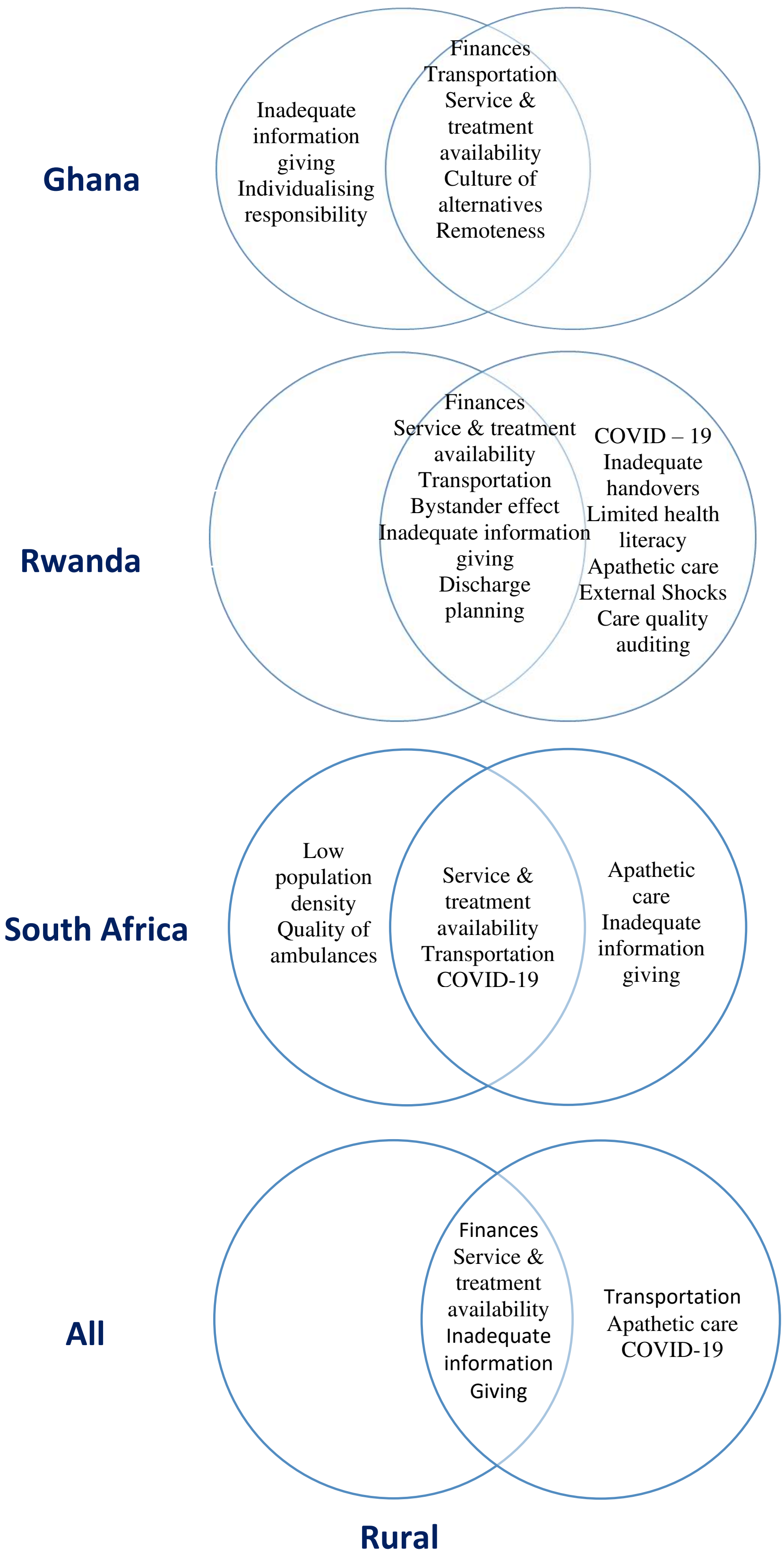

Appendix 3. Facilitators to good patient experience of trauma care, in descending order of frequency

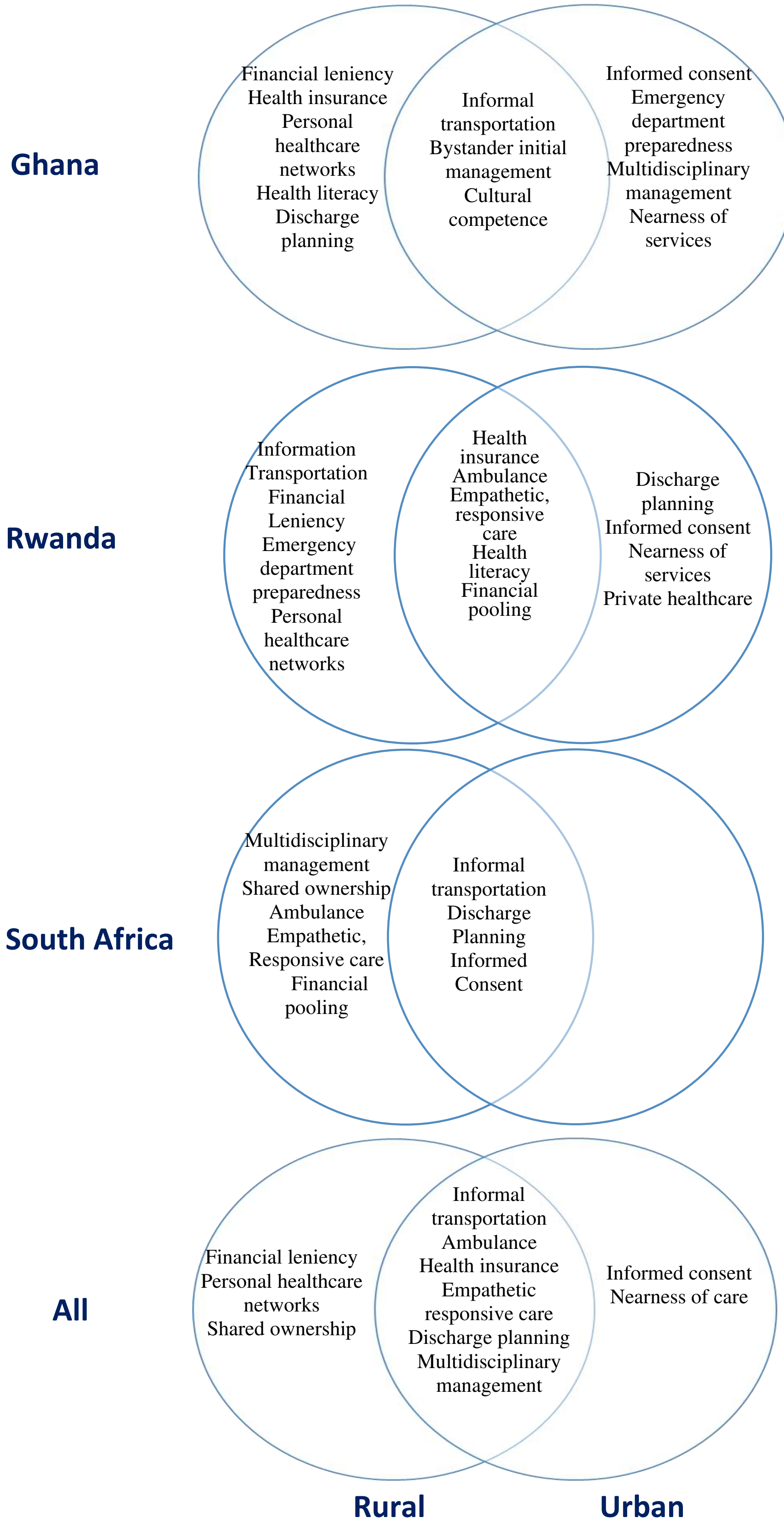

Appendix 4: Barriers to a good patient experience of trauma care in Ghana

	Ghana			
Barriers	Rural	Urban	Rural - Urban	Total
Financial Scarcity and inadequate healthcare coverage	10	9	1	19
Service and treatment availability	8	8	0	16
Transportation Barriers	7	8	-1	15
Cultural Value of alternative services	7	5	2	12
Inadequate Information Giving	7	3	4	10
Limited Health Literacy	2	8	-6	10
Apathetic Care	2	6	-4	8
Low Population Density	2	1	1	3
Narratives of Self-blame and Individualising Responsibility	2	0	2	2
Absence of care quality assessment	0	1	-1	1
COVID-19	0	1	-1	1
Absence of occupational health assessment	0	0	0	0
Bystander Effect	0	0	0	0
Care responsibilities	0	0	0	0
Clinician-patient power inequality	0	0	0	0
Delayed presented due to perceived low severity	0	0	0	0
Diagnostic uncertainty	0	0	0	0
Ethnic inequities in health system provision	0	0	0	0
Fear of surgery	0	0	0	0
Hospital catchment area system	0	0	0	0
Inability to self-care / loss of autonomy	0	0	0	0
Inadequate administration of Follow-Ups	0	0	0	0
Inadequate analgesia	0	0	0	0
Inadequate Discharge Planning	0	0	0	0
Inadequate Handovers	0	0	0	0
Language Barriers	0	0	0	0
Limited Specialist Training	0	0	0	0
Loss to follow up	0	0	0	0
Obstruction	0	0	0	0
Poor communication with family	0	0	0	0
Quality of ambulances	0	0	0	0
suboptimal clinical management	0	0	0	0
Unmet psychological needs	0	0	0	0
Unresponsiveness to formal complaints	0	0	0	0

Appendix 5: Barriers to a good patient experience of trauma care in Rwanda

	Rwanda			
Barriers	Rural	Urban	Rural - Urban	Total
Financial Scarcity and inadequate healthcare coverage	9	8	1	17
Service and treatment availability	7	9	-2	16
Transportation Barriers	7	8	-1	15
COVID-19	3	8	-5	11
Apathetic Care	5	3	2	8
Inadequate Discharge Planning	4	3	1	7
Bystander Effect	3	3	0	6
Inadequate Information Giving	3	2	1	5
Inadequate administration of Follow-Ups	2	1	1	3
Limited Health Literacy	3	0	3	3
Inadequate Handovers	0	2	-2	2
Absence of occupational health assessment	2	0	2	2
Obstruction	0	1	-1	1
Language Barriers	1	0	1	1
Limited Specialist Training	1	0	1	1
Diagnostic uncertainty	1	0	1	1
Clinician-patient power inequality	1	0	1	1
Inability to self-care / loss of autonomy	1	0	1	1
Narratives of Self-blame and Individualising Responsibility	1	0	1	1
Quality of ambulances	1	0	1	1
Inadequate analgesia	1	0	1	1
Cultural Value of alternative services	1	0	1	1
Hospital catchment area system	0	0	0	0
Ethnic inequities in health system provision	0	0	0	0
Care responsibilities	0	0	0	0
Unmet psychological needs	0	0	0	0
Fear of surgery	0	0	0	0
Loss to follow up	0	0	0	0
Unresponsiveness to formal complaints	0	0	0	0
Poor communication with family	0	0	0	0
Absence of care quality assessment	0	0	0	0
suboptimal clinical management	0	0	0	0
Delayed presented due to perceived low severity	0	0	0	0
Low Population Density	0	0	0	0

Appendix 6: Barriers to a good patient experience of trauma care in South Africa

	South Africa			
Barriers	Rural	Urban	Rural - Urban	Total
Apathetic Care	4	9	-5	13
Inadequate Information Giving	4	8	-4	12
Service and treatment availability	4	8	-4	12
Transportation Barriers	4	4	0	8
COVID-19	4	3	1	7
Financial Scarcity and inadequate healthcare coverage	3	3	0	6
suboptimal clinical management	1	2	-1	3
Delayed presented due to perceived low severity	2	1	1	3
Inadequate analgesia	1	2	-1	3
Unresponsiveness to formal complaints	0	2	-2	2
Poor communication with family	1	1	0	2
Absence of care quality assessment	0	2	-2	2
Inability to self-care / loss of autonomy	1	1	0	2
Quality of ambulances	2	0	2	2
Low Population Density	2	0	2	2
Inadequate Discharge Planning	0	2	-2	2
Hospital catchment area system	0	1	-1	1
Ethnic inequities in health system provision	0	1	-1	1
Care responsibilities	0	1	-1	1
Unmet psychological needs	0	1	-1	1
Fear of surgery	0	1	-1	1
Loss to follow up	0	1	-1	1
Inadequate administration of Follow-Ups	0	1	-1	1
Obstruction	0	0	0	0
Language Barriers	0	0	0	0
Limited Specialist Training	0	0	0	0
Diagnostic uncertainty	0	0	0	0
Clinician-patient power inequality	0	0	0	0
Inadequate Handovers	0	0	0	0
Absence of occupational health assessment	0	0	0	0
Narratives of Self-blame and Individualising Responsibility	0	0	0	0
Bystander Effect	0	0	0	0
Limited Health Literacy	0	0	0	0
Cultural Value of alternative services	0	0	0	0

Appendix 7: Facilitators to a good patient experience of trauma care in Ghana

	Ghana			
Facilitators	Rural	Urban	Rural - Urban	Total
Informal transportation	6	8	-2	14
Bystander Initial Management	2	3	-1	5
Financial Leniency / Charity	4	0	4	4
Health Insurance	4	0	4	4
Information giving & informed consent	0	4	-4	4
Personal Healthcare Networks	3	0	3	3
Health Literacy	3	0	3	3
Ambulance	2	1	1	3
Cultural Competence	0	2	-2	2
Nearness of Care	0	2	-2	2
Emergency Department Preparedness	0	2	-2	2
Multidisciplinary Management	0	2	-2	2
Community or Familial Financial Pooling	0	1	-1	1
Discharge Planning	1	0	1	1
Preferential Treatment	0	0	0	0
Police-facilitated transport	0	0	0	0
Communications Network	0	0	0	0
Familial Rehabilitative Support	0	0	0	0
Patient autonomy	0	0	0	0
NGO Support	0	0	0	0
Provision of mobility aids	0	0	0	0
Requesting Timely Care	0	0	0	0
Private Healthcare	0	0	0	0
Shared ownership of management plan	0	0	0	0
Empathetic Responsive care	0	0	0	0

Appendix 8: Facilitators to a good patient experience of trauma care in Rwanda

	Rwanda			
Facilitators	Rural	Urban	Rural - Urban	Total
Health Insurance	8	9	-1	17
Ambulance	6	8	-2	14
Information giving & informed consent	4	8	-4	12
Informal transportation	9	3	6	12
Empathetic Responsive care	4	6	-2	10
Health Literacy	4	5	-1	9
Community or Familial Financial Pooling	3	4	-1	7
Financial Leniency / Charity	5	2	3	7
Bystander Initial Management	2	4	-2	6
Discharge Planning	0	5	-5	5
NGO Support	2	1	1	3
Communications Network	1	1	0	2
Nearness of Care	0	2	-2	2
Private Healthcare	0	2	-2	2
Multidisciplinary Management	1	1	0	2
Preferential Treatment	1	0	1	1
Patient autonomy	0	1	-1	1
Cultural Competence	0	1	-1	1
Emergency Department Preparedness	1	0	1	1
Requesting Timely Care	1	0	1	1
Personal Healthcare Networks	1	0	1	1
Police-facilitated transport	0	0	0	0
Familial Rehabilitative Support	0	0	0	0
Provision of mobility aids	0	0	0	0
Shared ownership of management plan	0	0	0	0

Appendix 9: Facilitators to a good patient experience of trauma care in South Africa

	South Africa			
Facilitators	Rural	Urban	Rural - Urban	Total
Informal transportation	7	9	-2	16
Multidisciplinary Management	8	2	6	10
Discharge Planning	5	4	1	9
Empathetic Responsive care	7	1	6	8
Information giving & informed consent	4	4	0	8
Ambulance	6	1	5	7
Shared ownership of management plan	5	0	5	5
Provision of mobility aids	3	0	3	3
Community or Familial Financial Pooling	3	0	3	3
Requesting Timely Care	2	1	1	3
Private Healthcare	2	1	1	3
Familial Rehabilitative Support	1	1	0	2
Bystander Initial Management	1	1	0	2
Police-facilitated transport	1	0	1	1
Patient autonomy	1	0	1	1
Emergency Department Preparedness	1	0	1	1
Personal Healthcare Networks	1	0	1	1
Financial Leniency / Charity	1	0	1	1
Health Insurance	1	0	1	1
Preferential Treatment	0	0	0	0
Communications Network	0	0	0	0
Cultural Competence	0	0	0	0
NGO Support	0	0	0	0
Nearness of Care	0	0	0	0
Health Literacy	0	0	0	0

Appendix 10. ***Thematic corroboration of results with the findings of the prior mixed-methods study in Ghana, Rwanda and South Africa.***

Barriers	Barriers (Odland et al.)	Facilitators	Facilitators (Odland et al.)
Absence of care quality assessment	Present	Ambulance	Present
Absence of occupational health assessment		Bystander Initial Management	Present
Apathetic Care		Communications Network	
Bystander Effect	Present	Community or Familial Financial Pooling	
Care responsibilities		Cultural Competence	Present
Clinician-patient power inequality	Present	Discharge Planning	Present
COVID-19	Present	Emergency Department Preparedness	Present
Cultural Value of alternative services	Present	Empathetic Responsive care	Present
Delayed presented due to perceived low severity		Familial Rehabilitative Support	
Diagnostic uncertainty		Financial Leniency / Charity	
Inequities in health system provision	Present	Health Literacy	Present
Fear of surgery		Health Insurance	Present
Individual financial scarcity and inadequate health insurane coverage	Present	Informal transportation	
Hospital catchment area system	Present	Information giving and informed consent	Present
Inability to self-care / loss of autonomy		Multidiscliplinary Management	Present
Inadequate administration of Follow-Ups	Present	Nearness of Care	Present
Inadequate analgesia	Present	NGO Support	
Inadequate Discharge Planning	Present	Patient autonomy	Present
Inadequate Handovers		Personal Healthcare Networks	
Inadequate Information Giving	Present	Police-facilitated transport	
Language Barriers		Preferential Treatment	
Service and treatment availability	Present	Private Healthcare	
Health literacy	Present	Provision of mobility aids	
Limited Specialist Training		Requesting Timely Care	
Low Population Density	Present	Shared ownership of management plan	Present
Narratives of Self-blame and Individualising Responsibility			Coinciding themes (%)
Obstruction			52
Poor communication with family			
Quality of ambulances	Present		
Suboptimal clinical management			
Transportation Barriers	Present		
Unmet psychological needs	Present		
Unresponsiveness to formal complaints	Present		
Loss to Follow Up			
	Coinciding themes (%)		
	60.60606061		
