# Supplementary material for: Commonalities and differences in injured patient experiences of accessing and receiving quality injury care: a qualitative study in three sub-Saharan African countries
Source: BMJ Open. 2024 Jul 1;14(7):e082098. doi: 10.1136/bmjopen-2023-082098 (PMC11218010; doi:10.1136/bmjopen-2023-082098)
Supplement: Author's manuscript [file bmjopen-2023-082098.draft_revisions.pdf]

BMJ Open is committed to open peer review. As part of this commitment we make the peer review history of every article we publish publicly available.

When an article is published we post the peer reviewers' comments and the authors' responses online. We also post the versions of the paper that were used during peer review. These are the versions that the peer review comments apply to.

The versions of the paper that follow are the versions that were submitted during the peer review process. They are not the versions of record or the final published versions. They should not be cited or distributed as the published version of this manuscript.

BMJ Open is an open access journal and the full, final, typeset and author-corrected version of record of the manuscript is available on our site with no access controls, subscription charges or pay-per-view fees (http://bmjopen.bmj.com).

If you have any questions on BMJ Open's open peer review process please email info.bmjopen@bmj.com

Commonalities and differences in patient experiences of trauma systems in three sub-Saharan African countries

Journal:	*BMJ Open*
Manuscript ID	bmjopen-2023-082098
Article Type:	Original research
Date Submitted by the Author:	14-Nov-2023
Complete List of Authors:	The Equi-Trauma Collaborative , The Equi-Trauma Collaborative ; University of Birmingham College of Medical and Dental Sciences Ignatowicz, Agnieszka ; University of Birmingham Institute of Applied Health Research
Keywords:	Quality in health care < HEALTH SERVICES ADMINISTRATION & MANAGEMENT, Patient-Centered Care, QUALITATIVE RESEARCH

SCHOLARONE™
Manuscripts

I, the Submitting Author has the right to grant and does grant on behalf of all authors of the Work (as defined in the below author licence), an exclusive licence and/or a non-exclusive licence for contributions from authors who are: i) UK Crown employees; ii) where BMJ has agreed a CC-BY licence shall apply, and/or iii) in accordance with the terms applicable for US Federal Government officers or employees acting as part of their official duties; on a worldwide, perpetual, irrevocable, royalty-free basis to BMJ Publishing Group Ltd ("BMJ") its licensees and where the relevant Journal is co-owned by BMJ to the co-owners of the Journal, to publish the Work in this journal and any other BMJ products and to exploit all rights, as set out in our licence.

The Submitting Author accepts and understands that any supply made under these terms is made by BMJ to the Submitting Author unless you are acting as an employee on behalf of your employer or a postgraduate student of an affiliated institution which is paying any applicable article publishing charge ("APC") for Open Access articles. Where the Submitting Author wishes to make the Work available on an Open Access basis (and intends to pay the relevant APC), the terms of reuse of such Open Access shall be governed by a Creative Commons licence – details of these licences and which Creative Commons licence will apply to this Work are set out in our licence referred to above.

Other than as permitted in any relevant BMJ Author's Self Archiving Policies, I confirm this Work has not been accepted for publication elsewhere, is not being considered for publication elsewhere and does not duplicate material already published. I confirm all authors consent to publication of this Work and authorise the granting of this licence.

For peer review only

Commonalities and differences in patient experiences of trauma systems in three sub-Saharan African countries

The Equi-Trauma Collaborative

(the full list of authors, their contributions, and affiliations are at the end of the manuscript)

Corresponding authors:

Dr Agnieszka Ignatowicz and Dr Ciaran Kennedy, Institute of Applied Health Research, University of Birmingham, Birmingham, B15 2TT, UK. E-mail: a.m.ignatowicz@bham.ac.uk

Acknowledgement

We would like to acknowledge the work of our co-author Mr Abul Malik Abul-Latif whose tireless work enabled the completion of this study. Abdul passed away in September 2023.

Abstract

Background: In low- and middle-income countries, injury represents a significant contributor to mortality. Ensuring timely and equitable access to quality injury care for patients who have been injured is essential for reducing death and disability, but little is known about how patients experience assessing injury care.

Methods: We conducted an analysis of interview data with patients and community leaders in Ghana, South Africa, and Rwanda to understand the commonalities and differences in patient experiences of injury care.

Results: We found five broad barriers and six broad facilitators to patients accessing quality injury care. Our findings suggest that patients experience similar broad barriers and facilitators to accessing and receiving care across both rural and urban contexts in these countries.

Conclusion: Understanding these commonalities and differences can inform the development of targeted interventions to improve access to injury care in these sub-Saharan African countries.

What is already known on this topic

- In low- and middle-income countries, injuries significantly contribute to mortality. Timely and equitable access to quality injury care is crucial for reducing death and disability, but little is known about how patients experience assessing injury care.

For peer review only - http://bmjopen.bmj.com/site/about/guidelines.xhtml

What this study adds

- Patients in Ghana, South Africa and Rwanda face similar broad barriers and facilitators to accessing injury care, both in rural and urban settings. There may be universal factors influencing patient perceptions, regardless of the country or healthcare system.

How this study might affect research, practice or policy

- Understanding the common barriers and facilitators to quality injury care can inform the development of strategies for improving patient experiences in sub-Saharan African countries.

Introduction

Approximately 90% of injury-related mortality occurs in low- and middle-income countries (LMICs). [1] Across the African continent, the past two decades have witnessed a near 50% rise in healthy life-years lost from road traffic incidents. [1] This increased prevalence of injury has significant implications for health system capacity and finances. [2] Whilst preventative measures are urgently needed to reduce injury prevalence, access to quality healthcare for injured individuals is also essential to save lives and prevent disability. [3,4] Unfortunately, the past 25 years have seen a critical underfunding of injury care, compared to other global health concerns. [5] The establishment of a *sustainable development goal* target (3.6) to halve global deaths and injuries from road traffic accidents by 2030, may help to redress this balance. [6] Realising this aim is predicated on ensuring *equitable* access to quality injury care. That is, addressing barriers to patients *seeking*, *reaching*, *receiving,* and *remaining* in healthcare after injury. [7,8] These barriers may otherwise result in delayed or forgone injury care, which are associated with avoidable mortality and disability after injury. [4]

Knowledge of barriers to accessing care after injury is limited [9] and has mostly been studied at the healthcare facility level; studies seldomly look across the whole patient pathway, from point of injury to discharge from care. [7] Studies which have assessed the whole pathway have identified multiple barriers across all delays, with the majority (around 60%) focussing on receiving quality care, leaving a substantial number related to the prehospital patient pathway. [8] Identifying how to increase equitable access to injury care will require a whole health system approach to injury research, not one solely focussed on the capacity for patients to receive acute care. [7,8]

Exploring patient experiences of injury and injury care along the whole care pathway can contribute towards a systems-level understanding of injury care. Utilising qualitative methodology enables movement beyond simply delineating barriers to in access to services, to assessing the *acceptability* of services and subjective facilitators of quality injury care. It also has the potential to elicit

For peer review only - http://bmjopen.bmj.com/site/about/guidelines.xhtml

contextually dependent solutions to access barriers. Exploring these perspectives across multiple LMICs and foregrounding their commonalities may afford opportunities to improve health across multiple nations through transferable interventions.

Methods

This analysis used qualitative data collected from the *Equi-Trauma* study conducted in Ghana, South Africa, and Rwanda. It is reported according to the COREQ (Consolidated criteria for reporting qualitative research) checklist [11].

Participants and sampling strategy

For this paper, we only included participants that had a musculoskeletal injury. Methods for the main study have been published elsewhere [8]. For the main study, convenience sampling was employed to select persons in communities who had been injured and accessed – or attempted to access – care for an injury which occurred in the previous six months. Where possible, purposive sampling was done to enable representation of individuals across ages, sexes, and injury types. Participants were recruited from a rural and urban regions of each country. Hospital lists and contact with community leaders were used to identify participants who had or had not presented to clinics or hospitals for their injuries. When injured patients were not able to participate (for example, after death or disability), friends or relatives present during the injury were invited to participate.

Data collection process

Face-to-face in-depth interviews were conducted between January and October 2021 in urban and rural settings in all three countries. Interviews were conducted at locations suitable for participants, including hospitals, clinics, or patients' homes. Participants were interviewed individually using open-ended questioning for up to one hour. Interviews were conducted in the participants' preferred language. Interviews were conducted by a male and female surgeon in Ghana (SW and MY), a male non-clinical researcher in Rwanda (PN), and two female junior doctors in South Africa (KF and SN), all native to the country in which data were collected Interviews were audio-recorded and then transcribed verbatim. If interviews were not conducted in English, translation was conducted by in-country researchers (KF, MY, PN, SN and SW) and checked by in-country investigators (KC, JCB, ST).

Data collection tools

All interviewers were trained by a senior member of the research team (AI) to ensure standardisation of data collection methods. The English version of the topic guide, before adaptation to local contexts,

For peer review only - http://bmjopen.bmj.com/site/about/guidelines.xhtml

is attached (append. 1). This was developed to enable a rich understanding of patients' experiences of injury care along the whole care pathway from initial injury to rehabilitation. The topic guide was based on the authors' experiences and knowledge of the literature, permitting interviewer discretion to explore themes that emerged in specific interviews. It was piloted, and interviewers received feedback from a qualitative methods expert (AI) on their interviewing technique and field notes after two preliminary interviews. In all three countries, data saturation was reached towards the last interviews, reflecting the complexity of contexts and richness of the data.

Analytical approach

Computer-assisted thematic content analysis was conducted using *QSR NVivo*. [10] Codes were generated inductively, focussing on perceived barriers and facilitators to accessing quality injury care. A sample of interview transcripts across three study countries was read to identify the initial set of codes by two co-authors (AI and CK) through serial discussions. This generated an initial coding framework that was discussed in an analysis meeting between members of the research team (AI, CK and JD) and then used to code all remaining interview transcripts. Codes were gradually built into broader categories, with final themes being selected through comparison across transcripts and through discussion amongst co-authors, resolving discrepancies. Barriers and facilitators were coded as such dependent on how the participant reported them. Hence, themes could have been reported both as a barrier and a facilitator, depending on if the participants reported them negatively or positively. Some themes with perceived substantial thematic overlap were paired as a result of the further discussions between the two main researchers (AI & CK). Paired themes were reported together.

After aggregating themes, barriers and facilitators that were shared across at least two countries were identified, as well as discordant themes that were prevalent in only one country. The patient-reported barriers and facilitators to good experience of trauma care for each country are available in appendices (append.2-3). We then corroborated our results with the findings of our prior mixed-methods study in Ghana, Rwanda and South Africa [8]. Finally, we developed a data-informed causal loop diagram to demonstrate the effects of prominent barriers to seeking care and their impact on future outcomes.

Description of sample

A total of 60 in-depth interviews were conducted across Ghana, Rwanda, and South Africa, evenly segmented by urban versus rural areas. Ghanaian interviews were held in the Northern regional capital of Tamale and rural Yendi district (three female and 17 male injured individuals). Rwandan

For peer review only - http://bmjopen.bmj.com/site/about/guidelines.xhtml

interviews were held in the capital city of Kigali and rural Burera district of the Northern Province (five female and 15 male injured individuals). South African interviews were held in the urban township Khayelitsha and across the rural Western Cape (four female and 16 male injured individuals). Most of the persons interviewed in the main study had experienced musculoskeletal injuries and were eligible for inclusion into this study. One South African respondent in a rural setting was excluded from this analysis due to the mechanism of injury (burns). This left 59 interviews in the final analysis.

Ethical approval

Data collection was approved by: the Ghana Health Service Ethics Review Committee (GHS-ERC005/02/20); The Stellenbosch University Health Research Ethics Committee for South Africa (N20/01/010) and the Internal Research Board of the College of Medicine and Health Sciences of the University of Rwanda for Rwanda (No 050/CMHS IRB/2020). Additional approval was obtained from the Western Cape Department of Health (WC_202006_022) in South Africa, and from the respective hospitals before visiting the facilities. The overall study was approved by the University of Birmingham Research Ethics Committee, UK (ERN_20-00880). Funding was provided by the National Institute of Health Research, NIHR, award number 130036.

Authors' reflexivity statement

The Equi-Trauma Collaborative was a research team comprising academics, researchers and clinicians based in the UK, Rwanda, Ghana and South Africa. The team members had diverse cultural backgrounds. CK, AI, JD, MLO, AB, AH and JW represented high-income country researchers. The knowledge of lead investigators (KC, JC, ST) and involvement of local researchers in each study country have been crucial to the development and success of this project. All research staff who engaged in data collection are acknowledged as authors; specific roles of authors are outlined in this methods section and acknowledgments. The NIHR grant that funded this project was used to support the local research teams to undertake data collection, purchase qualitative analytical software, and disseminate the findings. Multiple virtual meetings were held with all research partners to train staff to undertake data collection, analyse the data and write up the results. There are three other manuscripts published as a results of this research, two of which are first authored by the early career researchers from Rwanda and South Africa. The manuscript from Ghana is currently in preparation.

The findings from this research formed the basis of an application for a larger grant that has been successful in securing the funding (Equi-injury: NIHR Global Health Group on Equitable Access to Quality Health Care for Injured People in Four Low or Middle Income Countries). The project is co-led

For peer review only - http://bmjopen.bmj.com/site/about/guidelines.xhtml

by the PIs based in the UK and South Africa. With small changes to the team in the UK and addition of another partner country (Pakistan), we have formed the Equi-injury Group. There is a large capacity building component attached to this project, which focuses on building sustainable partnerships and South-South learning.

Results

All individuals approached agreed to participate in the study with an attrition rate of 0%. The themes '*individual financial scarcity*' and *'inadequate health insurance coverage'; 'empathetic care'* and *'responsive care'*, and *'information giving'* and *'informed consent'* were paired by researchers (AI & CK) due to perceived substantial thematic overlap during coding. The themes aggregated were: '*health literacy*' and '*information giving*', '*informal transportation*' and '*ambulance transportation*', '*multidisciplinary management*' and '*discharge planning*'.

A total of 34 unique barriers and 25 unique facilitators to accessing perceived quality injury care were identified across the three countries (Tables 1 and 2). In Ghana there were 11 barriers and 14 facilitators; in Rwanda, 22 barriers and 21 facilitators; and in South Africa 23 barriers and 19 facilitators. Barriers and facilitators by rural or urban setting per country are presented in appendices (append.4-9). Direct quotations are shown for the most prevalent barriers common to each country and the most prevalent facilitators common to each country.

Table 1: Perceived barriers to a good patient experience of trauma care across Ghana, Rwanda & South Africa. The number refers to the number of times the barrier was reported. The most prevalent barriers are in red.

**Barriers**	**All Countries Total number**	**Rural**	**Urban**
**Service and treatment availability**	44	19	25
**Financial scarcity and inadequate healthcare coverage**	42	22	20
**Transportation Barriers**	38	18	20
**Apathetic Care**	29	11	18
**Inadequate Information Giving**	27	14	13
**COVID-19**	19	7	12
**Cultural Value of alternative services**	13	8	5
**Limited Health Literacy**	13	5	8
**Inadequate Discharge Planning**	9	4	5
**Bystander Effect**	6	3	3
**Low Population Density**	5	4	1
**Inadequate administration of Follow-Ups**	4	2	2
**Inadequate analgesia**	4	2	2
**Quality of ambulances**	3	3	0

For peer review only - http://bmjopen.bmj.com/site/about/guidelines.xhtml

Narratives of Self-blame and Individualising Responsibility	3	3	0
Inability to self-care / loss of autonomy	3	2	1
Delayed presented due to perceived low severity	3	2	1
Suboptimal clinical management	3	1	2
Absence of care quality assessment	3	0	3
Poor communication with family	2	1	1
Absence of occupational health assessment	2	2	0
Unresponsiveness to formal complaints	2	0	2
Inadequate Handovers	2	0	2
Loss to follow up	1	0	1
Limited Specialist Training	1	1	0
Diagnostic uncertainty	1	1	0
Language Barriers	1	1	0
Clinician-patient power inequality	1	1	0
Hospital catchment area system	1	0	1
Care responsibilities	1	0	1
Unmet psychological needs	1	0	1
Fear of surgery	1	0	1
Obstruction	1	0	1
Ethnic inequities in health system provision	1	0	1

Table 2: Perceived facilitators to a good patient experience of trauma care across Ghana, Rwanda & South Africa. The number refers to the number of times the barrier was reported. The most prevalent barriers are in red.

Facilitators	All Countries		
	Total number	Rural	Urban
Informal transportation	42	22	20
Information giving & informed consent	24	8	16
Ambulance	24	14	10
Health Insurance	22	13	9
Empathetic Responsive care	18	11	7
Discharge Planning	15	6	9
Multidisciplinary Management	14	9	5
Bystander Initial Management	13	5	8
Health Literacy	12	7	5
Financial Leniency / Charity	12	10	2
Community or Familial Financial Pooling	11	6	5
Private Healthcare	5	2	3
Personal Healthcare Networks	5	5	0
Shared ownership of management plan	5	5	0
Nearness of Care	4	0	4
Emergency Department Preparedness	4	2	2
Requesting Timely Care	4	3	1

For peer review

**Cultural Competence**	3	0	3
**NGO Support**	3	2	1
**Provision of mobility aids**	3	3	0
**Communications Network**	2	1	1
**Familial Rehabilitative Support**	2	1	1
**Patient autonomy**	2	1	1
**Preferential Treatment**	1	1	0
**Police-facilitated transport**	1	1	0

Common Barriers

Five broad barriers were shared and prevalent across at least two countries (Table 1). Less prevalent barriers are reported with their thematic frequencies in appendices (append.4-6)

Paired theme: individual financial scarcity & inadequate health insurance coverage

These themes were present 19 times in Ghanaian interviews, 17 times in Rwandan interviews and six times in South African interviews.

Participants struggled to finance transportation to hospitals (including ambulance services), medical care, pharmaceutical costs, and follow-up expenses. Respondents in Rwanda noted the selling of wealth (such as livestock and arable land) to fund medical treatment, whilst respondents from Ghana noted foregoing medical care due to financial barriers. In South Africa, unsuccessful disability and/or unemployment benefit applications resulted in financial barriers to follow-up care for some. Inadequate healthcare coverage affected numerous Ghanaian respondents who reported to be either uninsured or holding lapsed health insurance. Some Rwandan respondents reported that the tiered nature of their health insurance system provided inadequate coverage of certain healthcare services (e.g. one polytrauma patient received management for their musculoskeletal injury but not their dental trauma).

I. Service and treatment availability

This broad theme was coded 16 times in Ghanaian interviews, 16 times in Rwandan interviews and 12 times in South African interviews.

Some respondents noted an absence of adequate care in ambulances. On arrival at hospital, a lack of acute care beds and the lack of capacity to see patients in a timely manner was noted. Slow initial reviews were reportedly contributed to by inadequate staffing levels (particularly during religious and cultural festivals) and escalating patients to a more specialised hospital before providing initial management. The unavailability of essential services (such as blood transfusion and medical imaging) at certain hospitals in Ghana sometimes necessitated referrals. This service access barrier was

For peer review only - http://bmjopen.bmj.com/site/about/guidelines.xhtml

For peer review only

exacerbated by medical devices being in disrepair (e.g., a computerised tomography (CT) scanner). Other services in Ghana were reportedly not offered, including physiotherapy and occupational therapy. Respondents in Rwanda noted that some hospitals had low medicinal stock and placed the onus on patients to purchase their in-patient medications from pharmacies - sometimes situated at a distance from the hospital. Respondents in South Africa identified the lack of provision of weekend theatre lists and the tendency to postpone operations after a full day of fasting.

II. Transportation barriers

This broad theme was coded 15 times in interviews from Ghana, 15 times in interviews from Rwanda and eight times in interviews from South Africa.

Many respondents relied on the whims of passing vehicles to reach definitive care. Some respondents in rural Ghana noted this opportunistic transport seldom arrived due to the low population density, whilst some respondents in Rwanda stated that it was not feasible to use opportunistic motorcycles due to the degree of injury. Transportation was sometimes further delayed for Rwandan respondents due to the requirements of thorough police investigations.

Problematic environments impinged on transport arriving to the scene of injury and subsequently, definitive care. Untarmacked roads were reported in rural Ghana whilst damaged roads and inaccessible, hilly terrains were noted in rural Rwanda. Respondents in urban Ghana and Rwanda noted transport delays due to traffic congestion, exacerbated by reports of drivers not giving way to ambulances in Ghana.

There was consensus amongst most urban and rural Ghanaian respondents that there were no ambulances available for use, whilst Rwandan and South African respondents noted ambulances were often inexplicably delayed. Respondents in urban South Africa reported their area was not serviced by ambulances due to impenetrably dense shacks and/or ambulance staffs' fears of robbery. Rural South African respondents noted that ambulances operated like a taxi services, collecting multiple patients from the scene of each injury before reaching definitive care.

Many patients were referred to multiple facilities from low to highly specialised centres for treatment, until an appropriate hospital for their injury was reached – each escalation necessitating further transport. Rwandan respondents noted the scarcity of ambulances for inter-hospital transfers. Ghanaian respondents noted that the crowdedness of public transport (notably buses) led to some individuals not seeking follow-up care. Some Rwandan interviewees reported reaching follow-up by foot due to a lack of public transport, exacerbated by COVID-19-associated road and public transport closures.

III. Apathetic care

For peer review only

This theme was coded eight times in Ghanaian interviews, eight times in Rwandan interviews and 13 times in South African interviews. In Ghanaian and South African interviews, reported apathetic care skewed towards the urban sample, whereas in Rwandan interviews apathetic care skewed marginally towards rurality.

Some respondents from Ghana noted they remained unattended to by healthcare professionals for hours after arriving at the final health facility. Moreover, those needing higher level care often received no care or human interaction from healthcare professionals prior to inter-facility transfer. Some respondents from Ghana noted they remained unattended to by healthcare professionals for hours at the time of initial presentation. Moreover, those earmarked for escalation to a more specialist unit often received no care or human interaction from healthcare professionals prior to transfer. Respondents from Ghana also noted that doctors often delegated all patient interaction to the nursing staff. Both Ghanaian and Rwandan respondents noted that nursing staff were generally inattentive to patients in pain, whilst respondents in South Africa stated nursing staff were unresponsive to patient needs throughout the nightshift. Respondents in Rwanda and South Africa stated that healthcare professionals could sometimes become quarrelsome or aggressive when patients made enquiries regarding their care. An interviewee from South Africa felt that healthcare professionals were in their respective roles purely for financial reasons and not because they cared about their patients or profession. One Ghanaian respondent reported that healthcare professionals did not explore patients' feelings, concerns, or psychological aspects of injury.

Aggregated theme: Health literacy & information giving

Health literacy (as compared against authors' knowledge of health services) was coded 10 times in Ghanaian interviews, three times in Rwandan interviews and was not coded in South African interviews. Inadequate information giving was coded 10 times in Ghanaian interviews, five times in Rwandan interviews, and 12 times in South African interviews. These themes were aggregated after coding.

Some Ghanaian respondents reported that injured patients were taken to their family homes to deliberate on next steps, rather than directly to definitive care. Health centres were often used as the first port-of-call after injury as opposed to hospitals due to their relative proximity. Some Ghanaian respondents reported that hospital care was reserved for those with head injuries, loss of consciousness, substantial bleeding, or soft tissue injury. Other Ghanaian interviewees reported that hospitals were inappropriate for fracture management and medical intervention may lead to limb deformity or amputation. These individuals perceived that injury patients would be spared from these complications by using local, bone-setting services. One Rwandan respondent stated that ambulance

For peer review only - http://bmjopen.bmj.com/site/about/guidelines.xhtml

For peer review only

transportation was reserved for pregnancies, whilst another stated that an ambulance would not have been appropriate for a patient with a fractured arm as they had the capacity to walk.

Some Ghanaian respondents stated that clinicians did not provide them with any information regarding their diagnosis, management plan or expected fit-to-work date. Some Rwandan respondents noted no explanations were provided for their early discharge, their follow-up plan or why they had no follow-up appointments scheduled. South African respondents reported that there was no information given on how to access inter-hospital transportation or their self-care requirements. One South African respondent noted no explanation was offered regarding their surgical error.

Common facilitators

Six broad facilitators were shared and prevalent across at least two countries (Table 2). Less prevalent facilitators are only reported with their thematic frequencies in appendices (append.7-9).

Aggregated theme: Informal transportation & ambulance transportation

These two themes were aggregated after coding. Informal transportation was coded 14 times in Ghanaian interviews, 12 times in Rwandan interviews and 16 times in South African interviews. Ambulance transportation was coded three times in Ghanaian interviews, 14 times in Rwandan interviews and seven times in South African interviews.

Respondents from all three countries relied heavily on passing-by motorcycles, tricycles, and cars to reach definitive care. Reasons for seeking this form of transportation included its ready availability, its timeliness to arrival at (and its proximity to) the site of injury, its relative affordability, and local norms. Ghanaian respondents noted that injured patients' family members often used their own vehicles, borrowed vehicles, or sought known drivers in the region to transport their relative to emergency care. Transportation of injured Rwandan respondents was sometimes facilitated by the police, the army, or by a doctor. In rural Rwandan settings that were inaccessible to vehicles, transportation was afforded using traditional stretchers known as "ingobyi". Some South African respondents reported driving themselves to acute care or being transported by their employer. Informal transportation was also employed for inter-hospital transfers and reaching follow-up appointments. South African respondents noted utilising friends, family, and neighbours to reach follow-up care, whilst participants across the three countries utilised public transportation (including bus and taxis) to reach follow-up care.

Many Rwandan and rural South African respondents utilised ambulances to reach emergency care, with some Rwandan respondents also utilising ambulances for inter-hospital transfers. Numerous

For peer review only - http://bmjopen.bmj.com/site/about/guidelines.xhtml

Rwandan respondents commented favourably on the timeliness and availability of ambulances. South African respondents often noted police-facilitation of ambulance requests.

I. Information giving and informed consent

These themes were paired prior to coding and were coded four times in Ghanaian interviews, 12 times in Rwandan interviews and eight times in South African interviews.

Ghanaian and South African respondents noted that clinical staff informed them about their diagnosis and imaging results. Their proposed management plans, including the regularity of bandage redressing, medications indicated and required surgical procedures were also explained. Respondents from Rwanda, particularly those in an urban setting, commented on the time given to patients to deliberate on treatment decisions, together with clinicians' support of patients' refusal of treatment after initial consent. Respect of patients' preference in the decision-making process with periodic clarification of understanding was also noted amongst Rwandan respondents.

II. Health insurance

This theme was coded four times in Ghana, 17 times in Rwanda and once in South Africa.

The utilisation of health insurance in Rwanda was common in interviews, whereas its utilisation in South Africa was reported once. Across all countries health insurance was noted to increase accessibility to healthcare services as well as the range of healthcare services available. Health insurance enabled extended in-patient stay, whilst making out-of-pocket payments manageable. In Rwanda, certain types of insurance were considered more beneficial than others, including "*Community-Based Health Insurance"* and *'RAMA'* (a civil service insurance scheme). Charity also played a role in insurance provision in rural Rwanda, most notably *Partners in Health,* which was active in the Burera area when the rural study was conducted.

III. Aggregated theme: Multidisciplinary management & discharge planning

Multidisciplinary management was coded twice in Ghanaian interviews, twice in Rwandan interviews and 10 times in South African interviews. Discharge planning was coded once in Ghanaian interviews, five times in Rwandan interviews and nine times in South African interviews. These themes were aggregated after coding.

One Ghanaian respondent noted that physiotherapists provided them with a *'Zimmer'* frame for physical rehabilitation, whilst a Rwandan respondent noted that a physiotherapist attended their home and built equipment to practise mobilising. Physical therapy featured in many South African interviews, with activities including resistance band training and home exercises. This involved both in-patient and out-patient therapy, often on a regular basis. One South African respondent noted they received occupational therapy input too.

Rwandan respondents in an urban setting and South African respondents in both settings mentioned

For peer review only - http://bmjopen.bmj.com/site/about/guidelines.xhtml

discharge planning. The Rwandan respondents noted that planned follow-up appointments were arranged for patients prior to discharge. Successive appointments enabled doctors to monitor progress (assisted by imaging) and address issues through prescribing and nurse-led wound redressing. South African respondents received clinician follow-up as well as specific dates for stitch/cast removal, wound redressing, and physiotherapy.

IV. Paired theme: Empathetic & responsive care

These themes were paired prior to coding and were coded twice in Ghanaian interviews, 10 times in Rwandan interviews and eight times in South African interviews. One Ghanaian respondent noted that healthcare professionals displayed a gentle manner, explored patients' feelings and showed concern for their work, whilst another noted that doctors tried to inspire their patients. Rwandan respondents noted that healthcare professionals were responsive to their needs. This included providing analgesia in response to pain, allowing a carer to stay at a patient's bedside overnight and addressing and alleviating a patient's concern about the possibility of amputation. One Rwandan respondent commented on the provision of a counsellor who would converse with patients and assess their wellbeing. Respondents stated that healthcare professionals did not complain of tiredness, spoke with patients throughout procedures, with one healthcare professional accompanying a patient on their hospital transfer.

South African respondents in a rural setting noted that healthcare professionals checked patients' condition regularly, communicated in an understanding manner, provided bedpans for those who struggled to reach the toilet and regularly changed sheets in response to bleeding.

V. Health literacy

This theme was coded three times in Ghanaian interviews, nine times in Rwandan interviews and was not coded in South African interviews.

Rwandan respondents (in contrast to Ghanaian respondents) primarily utilised hospital over bone-setting services after acute injury, implying that this was the norm in Rwanda. Respondents noted that in cases where an individual bystander suggested traditional methods, they tended to be overruled by group consensus. One Rwandan interviewee noted that traditional healing may lead to long-term complications, which would necessitate medical management. A Rwandan respondent suggested that ambulance care was ideal as ambulances were given priority access in traffic, whilst another eluded to the benefit of initial patient management in an ambulance.

Discordant data

Two barriers and one facilitator were identified that were prevalent in one country only. The barrier, '*cultural value of alternative services*' was coded 12 times in Ghanaian interviews - seven times in rural

For peer review only - http://bmjopen.bmj.com/site/about/guidelines.xhtml

settings and five times in urban settings. The barrier '*bystander effect'* was coded six times in Rwandan interviews, evenly distributed between rural and urban settings. The facilitator *'shared ownership of management plan'* was coded five times in rural South African interviews.

Rural versus urban findings

Across all three countries, the most prevalent barriers in urban settings tended to be mirrored in rural settings *Service and treatment availability* was coded 20 times in urban settings and 19 times in rural settings, *individual financial scarcity and inadequate health insurance coverage* was coded 20 times in urban settings and 22 times in rural settings, and *inadequate information giving* was coded 13 times in urban settings and 14 times in rural settings. Two prevalent barriers were shared amongst urban and rural settings but more frequent in urban areas: *transportation barriers* were coded 25 times in urban settings and 18 times in rural settings, whilst *apathetic care* was coded 18 times in urban settings and 11 times in rural settings.

Across all three countries, the most prevalent facilitators in urban settings were also prevalent in rural settings. *Informal transportation* was coded 20 times in urban settings and 22 times in rural settings, *Ambulance transportation* was coded 10 times in urban settings and 14 times in rural settings, *Health insurance* was coded nine times in urban settings and 13 times in rural settings, *Empathetic & Responsive Care* was coded nine times in urban settings and 11 times in rural settings, and *Discharge planning* was coded 9 times in urban settings and six times in rural settings. One prevalent facilitator skewed towards urbanity whilst another skewed towards rurality: *Information giving and informed consent* was coded 16 times in urban settings and eight times in rural settings, whilst *financial leniency or charity* was coded twice in urban settings and nine times in rural settings. This latter theme involved examples of ambulances not charging for transportation, clinicians not charging acute injury care and physiotherapists not charging outpatient care for individuals with limited financial resources in Ghana and Rwanda.

We constructed a causal loop diagram based on data for Ghana and Rwanda (but not for South Africa due to reportedly better health care coverage) focussing on the issues of lack of payment and suboptimal clinical management as these were the two most prominent barriers captured in the analysis. This shows the impoverishing cycle of requirements to pay for care, which either results in forgone care and impoverishment through increased disability and lack of ability to work, thence inability to afford future healthcare insurance or paying for optimal care resulting in financial deprivation and inability to afford future healthcare insurance (Figure 1).

For peer review only - http://bmjopen.bmj.com/site/about/guidelines.xhtml

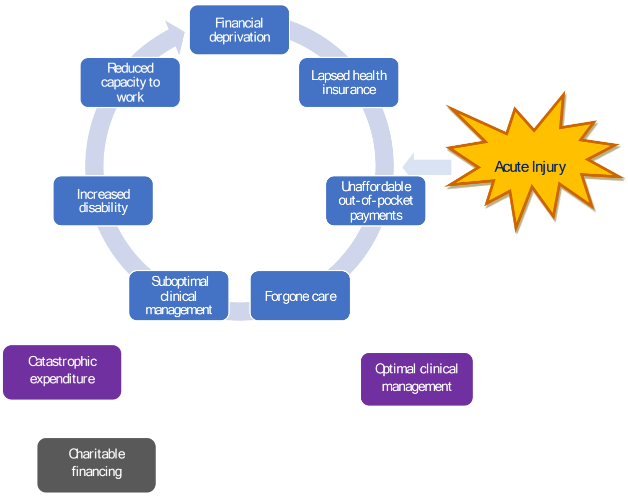

Figure 1. Cycle of disability and deprivation after acute injury.

Discussion

Our analysis shows that when examining patient experiences using qualitative research methods, broad barriers and facilitators to accessing injury care are comparable across contexts. The findings suggest that there are universal factors influencing patient perceptions, independent of the context or healthcare system, and emphasise the significance of understanding these barriers and facilitators to inform policy decisions and develop interventions to ensure equitable and patient-centred care in sub-Saharan African countries.

Five broad barriers and six broad facilitators to accessing quality injury care were identified. These themes were prevalent and shared across at least two countries but demonstrated inter-country and intra-country (between urbanity and rurality) variation in thematic frequency.

Individual financial scarcity and the associated theme of inadequate health insurance coverage were predominant barriers across all three countries. These barriers contributed to poorer patient experiences of injury care by erecting barriers to seeking, reaching, receiving, and remaining in healthcare. [21] They notably led to a lack of financial risk protection, which can increase the likelihood of injured patients forgoing care or opting for I/CHE. [22] Interestingly, the response of injured patients to a lack of financial risk protection appeared to vary between countries.

Amongst Ghanaian respondents, those without the capacity to pay tended to forgo care or incurring I/CHE. Forgone care may have led to many injured Ghanaians acquiring disability amenable to healthcare – that is, potentially preventable given timely, effective injury care. [4,23] Their disability

For peer review only - http://bmjopen.bmj.com/site/about/guidelines.xhtml

severity in turn may have affected their capacity to work and therefore finance future healthcare. In this vein, *Mock et al.* noted that 72% of injured Ghanaian patients reported a loss of income, primarily affected by the length of ensuant disability. [24] Whilst forgone care was reported amongst some Rwandan respondents in our study, others reported opting to sell, pawn or loan wealth (e.g., livestock and arable land) to fund acute injury care. This potentially I/CHE affected their capacity to afford future episodes of healthcare, forming another pathway to amenable disability. This has been shown in other areas of care, for example, in their study of caesarean section patients, *Niyigena et al.* found that many Rwandans seeking healthcare are exposed to I/CHE despite the provision of community health insurance. This is due to the insurance not covering healthcare-related transportation costs, costs of care-giving, follow-up costs or lost wages [25] - all alluded to in our study. Financial barriers to follow-up care were also reported by South African respondents in our study, however there were no reports of insurance inadequacy, I/CHE or forgone acute care for financial reasons. In fact, the relative infrequent coding of the facilitator, health insurance, in South African interviews likely reflects the presumed normality of healthcare coverage in this more mature health system. Interestingly, across the whole study population an association was identified between rurality and 'financial leniency or charity', pertaining to expenses associated with the injured patient care pathway. This was predominantly noted amongst Ghanaian and Rwandan respondents in rural settings (perhaps due to health insurance sufficiency in rural South Africa).

Sapkota et al. have previously described a vicious cycle of chronic disease and poverty in Nepal. They argued convincingly that in LMICs with significant out-of-pocket expenditure for healthcare, chronic disease and poverty become mutually reinforcing across the life course. [26] Herein, we have adapted this concept to describe a vicious cycle of disability and deprivation occurring after acute injury (fig. 1). Our cycle contains a primary pathway initiated by the injured patient's decision to forgo care (as exemplified by Ghanaian respondents), with a secondary pathway initiated by the decision to pursue I/CHE (as exemplified by Rwandan respondents). (A clinical vignette has been included which demonstrates this secondary pathway in action in appendices). A minor tertiary pathway is accessible to some through charitable funding of their acute injury care. Due to the contingent nature of this funding, it may not be accessible for future episodes of healthcare, potentially re-exposing individuals to unaffordable out-of-pocket payments.

Formal ambulance transportation systems did not enable all injury patients to reach definitive care, although informal methods provided some assistance. In our study, the dearth of ambulances was felt most strongly amongst Ghanaian respondents. It should be emphasised however that the reported access to ambulance services does not seem to translate into service uptake (at least in our small sample). Interestingly, modelling in a Cape Town emergency department has suggested that simply

For peer review only - http://bmjopen.bmj.com/site/about/guidelines.xhtml

increasing the number of ambulances available would result in rapidly diminishing improvements in ambulance response time - insufficient to meet national targets. [12] This resonates with respondents from urban Cape Town in our study, who suggest that adequate area servicing by ambulances may be more dependent on perceived safety of ambulance personnel than ambulance availability. Supporting the development of community-based transportation systems may also be more contextually appropriate, for example in rural Rwanda where traditional stretchers are used as the mountainous regions are impenetrable to ambulances. Such public engagements in health system reform in sub-Saharan Africa have however had a tendency towards tokenistic or gestural engagement rather than true participation. [13] This would need addressing if reform of injury transportation systems was considered.

Myriad problems pertaining to service and treatment availability were highlighted across all three countries. Respondents noted the absence of care giving at both the site of injury and during ambulance transit. These concerns are borne out in the literature. A recent review of pre-hospital airway management in 11 LMICs found that despite the provision of advanced life support training in many settings, first responders tended to only employ basic life support techniques for airway management. [14] The predilection for basic life support also emerged within hospital settings, with only 20% of physicians in a rural Ghanaian hospital providing advanced airway manoeuvres. [15]. While such research is informing, research in high income countries suggests that for certain types of injuries and for certain skill mixes of healthcare providers, the speed of transfer to a hospital may be more important than providing prolonged care at the scene. [16,17]. The importance of prehospital transfer time has also been shown in reducing maternal mortality in Sierra Leone. [18]

Once at definitive care, respondents noted the unavailability of essential services such as transfusion and imaging services. These issues were often further compounded by medicinal and dressing shortages. The absence of essential medicines in hospitals and affiliated pharmacies can contribute to increased out-of-pocket expenditure of injury patients (for both pharmaceuticals and the travel costs to dispensaries), which exposes patients to (impoverishing/catastrophic expenditure) I/CHE. '*Stockouts*' affect patient safety through increasing patient exposure to substandard and falsified (SF) medicines, which are estimated to form 13.6% of drug usage in LMICs. [19] They also lead to hospital underutilisation, poorer patient experiences of care, and distrust of healthcare professionals. [20] Whilst imaging and medication may ultimately be available in some hospitals and pharmacies respectively, they may not be affordable, hence their access will be less timely, affecting patient experiences and outcomes. In this respect, the availability of services and medicines and the affordability of episodes of healthcare lie on a continuum of access barriers to quality injury care.

For peer review only - http://bmjopen.bmj.com/site/about/guidelines.xhtml

Individuals in all three countries raised concerns with apathetic care, whilst episodes of empathetic care were commended. This variability is exemplified through the juxtaposition of a Ghanaian respondent, who explained that healthcare professionals did not explore patients' feelings, concerns or psychological aspects of injury, with a Rwandan respondent, who received a counsellor for precisely these reasons. Interactions with healthcare staff have significant implications for patients' perceptions of care quality, where studies have found that positive patient experiences are influenced by the disposition of doctors, their tone of voice, their attitudes towards patients and their attentiveness. [27] Awareness of cultural differences is also of great importance in an injury setting, where a heterogenous mix of patients present. Ghana is a country with significant internal migration, affecting 40% of the population, with rural-to-urban migrants experiencing linguistic, cultural, and financial Nigerian orthopaedic unit found that in almost one third of delayed operations there was not full concordance between doctors and patients on the reason for delay. [28] It is also important to recognise that ways of knowing (epistemologies) vary across different Global South settings, and the preference for bone-setting services (over trauma centres) amongst certain individuals in sub-Saharan Africa is based on long historical roots. [29] Thus any attempt to strengthen trauma systems should valorise rather than marginalise these traditional knowledges. It is worth emphasising that treatment pathways of individual injury patients are complex and often involve multiple transitions between traditional and medical services. [30] It may be more appropriate therefore to consider these services as two components of a single health system with opportunities for complementary work, rather than discrete competing treatment decisions. Indeed, a recent systematic review found that traditional bone setters were keen to engage with orthopaedic services and training courses in their management of musculoskeletal injury, which has been shown to reduce complications of bone-setting. [31] One option to increase access to quality trauma care through collaborative work could entail situating rehabilitative physiotherapy in bone-setting centres.

We note that some barriers were also framed as facilitators even, sometimes, by the same participants in interviews suggesting these are different sides of the same coin. This was especially the case for transportation, where informal transportation, when available is a facilitator, whereas when unavailable is seen as a barrier. Likewise for apathetic and empathetic care. Although many of these similar themes were reported more frequently as barriers than facilitators, these findings suggests that with deeper understanding of the context, it may be possible to turn the barriers into facilitators. For example, by formalising informal transport or by holding up examples of empathetic care and the benefits of this for others to learn from.

For peer review only - http://bmjopen.bmj.com/site/about/guidelines.xhtml

Limitations & strengths

The data collection for this study was conducted during periods of national COVID-19 lockdown, which may have transiently affected injury epidemiology. During their lockdown, South Africa introduced an alcohol ban, which limited exposure to a significant risk factor for injury. [32] The pandemic also precluded respondent validation of transcribed interviews. Moreover, we did not stratify our analyses by gender due to the small sample size of women recruited, which would have risked deanonymizing participants. These limitations have gender equity implications; however, we recognise also that most injuries are suffered by men, which is reflected by their dominance in our sample. [1] Whilst rural-urban divisions were explored in our study, large areas of each country were left unsampled, limiting national representativeness. The absence of ethnic coding of transcripts prohibited any investigation of ethnic inequities in injury care, which are known to exist for myriad health conditions. Further research on patients' experiences of injury care employing an *intersectional lens,* segmenting data by gender, ethnicity, and socioeconomic status, may illuminate any undocumented inequities.

A strength of our study was that its design was informed by prior stakeholder engagement in Ghana, Rwanda, and South Africa. [8,33] Community leaders were also consulted prior to the study and involved in various aspects of the research. The credibility of our study has been improved through using multiple analysts and through triangulating our findings with the published literature. Investigator checking was undertaken at multiple stages throughout the thematic coding process. Our prior mixed-methods study in Ghana, Rwanda and South Africa has been used for methodological and data source triangulation. 20/34 barriers (58.8%) in our study and 13/25 facilitators (52%) were identified in this prior study, (append. 10) strengthening the confidence in the validity of these findings. [8]

Conclusion

Despite past research indicating disparities between countries, this study demonstrates that when using qualitative research methods to examine patient experiences of trauma systems, broad barriers and facilitators to accessing injury care are comparable across contexts. Our findings suggest that there may be universal factors influencing patient perceptions regardless of the country or healthcare system and highlight the importance of understanding these common barriers and facilitators to develop effective strategies for improving patient experiences of injury care in sub-Saharan African countries.

For peer review only - http://bmjopen.bmj.com/site/about/guidelines.xhtml

Acknowledgements

We would like to acknowledge all participants in our study, who generously shared their time, experiences and insights with us.

Authors

Equi-Trauma Collaborative:

Ciaran Kennedy[1], Agnieszka Ignatowicz[1], Maria Lisa Odland[1,2,3,4,5], Abdul-Malik Abdul-Latif[1,6], Antonio Belli[7,8], Anthony Howard[9,10], John Whitaker[1,11], Kathryn M. Chu[12,13], Karen Ferreira[12], Eyitayo O. Owolabi[12], Samukelisiwe Nyamathe[12], Stephen Tabiri[14,15,16], Bernard Ofori[14], Sheba Mary Pognaa Kunfah[14], Mustapha Yakubu[15,17], Abebe Bekele[18,19], Barnabas Alyande[20,21], Pascal Nzasabimana[22], Jean-Claude Byiringiro[22,23] and Justine Davies[1,12,24]

1. Institute of Applied Health Research, University of Birmingham, Birmingham, UK

2. Department of Obstetrics and Gynaecology, St. Olavs University Hospital, Trondheim, Norway

3. Malawi-Liverpool-Wellcome Trust Research Institute, Blantyre, Malawi

4. Department of Public Health and Nursing, NTNU, Norwegian University of Science and Technology, Trondheim, Norway

5. Institute of Life Course and Medical Sciences, University of Liverpool, Liverpool, United Kingdom

6. Volta Regional Health Directorate, Ghana Health Service, Accra, Greater Accra, Ghana

7. Institute of Inflammation and Ageing, University of Birmingham, Birmingham, UK

8. National Institute for Health Research Surgical Reconstruction and Microbiology Research Centre, Birmingham, UK

9. Leeds Institute of Rheumatic and Musculoskeletal Medicine, School of Medicine, University of Leeds, Leeds, UK

10. Nuffield Department of Orthopaedics, Rheumatology and Musculoskeletal Sciences, National Institute of Health Research (NIHR) Biomedical Centre, University of Oxford, Headington, Oxford, UK

11. King's Centre for Global Health and Health Partnerships, King's College London Faculty of Life Sciences and Medicine, London, UK

12. Centre for Global Surgery, Department of Global Health, Stellenbosch University, Cape Town, South Africa

13. Department of Surgery, University of Botswana, Gaborone, Botswana

14. Ghana HUB of NIHR Global Surgery, Tamale, Ghana

For peer review only - http://bmjopen.bmj.com/site/about/guidelines.xhtml

15. Department of Public Health, Tamale Teaching Hospital, Tamale, Ghana

16. Department of Surgery, Tamale Teaching Hospital, Tamale, Ghana

17. School of Medicine and Health Sciences, University for Development Studies, Tamale, Ghana

18. University of Global Health Equity, Kigali, Rwanda

19. Department of Surgery, Addis Ababa University, Addis Ababa, Ethiopia

20. Center for Equity in Global Surgery, University of Global Health Equity, Kigali, Rwanda

21. Program in Global Surgery and Social Change, Harvard Medical School, Boston, Massachusetts, USA

22. University of Rwanda, Kigali, Rwanda

23. Department of Surgery, University Teaching Hospital of Kigali, Kigali, Rwanda

24. Medical Research Council/Wits University Rural Public Health and Health Transitions Research Unit, Faculty of Health Sciences, School of Public Health, University of the Witwatersrand, Johannesburg, South Africa

Contributors

CK conducted the analyses and wrote the initial draft. JD and AI developed the idea for this analysis and supervised CK in the analyses and writing. JD, KC and JC developed the protocol for the main study and oversaw it. JW and AH contributed ideas for the protocol and running of the project. MLO, AI, A-MA-L, AB, BA, JC, ST, KC and JD oversaw the day-to-day data collection and supervised the data collectors. KF, SN, BO, SMPK, MY and PN collected data for the study. All authors have reviewed and approved the manuscript. JD is the guarantor.

Funding

This research was funded by the National Institute for Health and Care Research grant number 130036.

Competing interests

None declared.

Patient and public involvement

Patients and/or the public were not involved in the design, reporting, or dissemination plans of this research.

Data availability statement

For peer review only - http://bmjopen.bmj.com/site/about/guidelines.xhtml

Data are available upon a reasonable request from Prof Justine Davies (email: j.davies.6@bham.ac.uk).

Patient consent for publication

Verbal and signed consent obtained directly from all participants.

Ethics approval

The overall study was approved by University of Birmingham Research Ethics Committee, UK (ERN_20-00880). Data collection in each individual country were approved by appropriate Ethics Review Boards: Ghana Health Service Ethics Review Committee (GHS-ERC005/02/20); The Stellenbosch University Health Research Ethics Committee for South Africa (Reference: N20/01/010) and the IRB of the College of Medicine and Health Sciences of the University of Rwanda for Rwanda (No 050/CMHS IRB/2020). Additional approval was obtained from the Western Cape Department of Health (Reference: WC_202006_022) in South Africa, and in the other countries approval was sought from the respective hospitals before visiting the facilities.

Provenance and peer review

Not commissioned; externally peer reviewed.

References

1 WHO. Injuries and violence factsheet. 2021.

2 Ryan-Coker MFD, Davies J, Rinaldi G, *et al.* Economic burden of road traffic injuries in sub-Saharan Africa: a systematic review of existing literature. *BMJ Open* 2021;**11**:e048231. doi:10.1136/bmjopen-2020-048231

3 Mock C, Joshipura M, Arreola-Risa C, *et al.* An Estimate of the Number of Lives that Could be Saved through Improvements in Trauma Care Globally. *World J Surg* 2012;**36**:959–63. doi:10.1007/s00268-012-1459-6

4 Edem IJ, Dare AJ, Byass P, *et al.* External injuries, trauma and avoidable deaths in Agincourt, South Africa: a retrospective observational and qualitative study. *BMJ Open* 2019;**9**:e027576. doi:10.1136/bmjopen-2018-027576

5 Hollis SM, Amato SS, Bulger E, *et al.* Tracking global development assistance for trauma care: A call for advocacy and action. *J Glob Health* 2021;**11**:04007. doi:10.7189/jogh.11.04007

6 WHO. SDG Target 3.6: Road Traffic Injuries.

For peer review only - http://bmjopen.bmj.com/site/about/guidelines.xhtml

7 Whitaker J, O'Donohoe N, Denning M, *et al.* Assessing trauma care systems in low-income and middle-income countries: a systematic review and evidence synthesis mapping the Three Delays framework to injury health system assessments. *BMJ Glob Health* 2021;**6**:e004324. doi:10.1136/bmjgh-2020-004324

8 Odland ML, Abdul-Latif A-M, Ignatowicz A, *et al.* Equitable access to quality trauma systems in low-income and middle-income countries: assessing gaps and developing priorities in Ghana, Rwanda and South Africa. *BMJ Glob Health* 2022;**7**:e008256. doi:10.1136/bmjgh-2021-008256

9 Whitaker J, Nepogodiev D, Leather A, *et al.* Assessing barriers to quality trauma care in low and middle-income countries: A Delphi study. *Injury* 2020;**51**:278–85. doi:10.1016/j.injury.2019.12.035

10 QSR International Pty Ltd. (2020) NVivo (released in March 2020), https://www.qsrinternational.com/nvivo-qualitative-data-analysis-software/home.

11 Tong A, Sainsbury P, Craig J. Consolidated criteria for reporting qualitative research (COREQ): a 32-item checklist for interviews and focus groups. *International Journal for Quality in Health Care* 2007;**19**:349–57. doi:10.1093/intqhc/mzm042

12 Stein C, Wallis L, Adetunji O. Meeting national response time targets for priority 1 incidents in an urban emergency medical services system in South Africa: More ambulances won't help. *South African Medical Journal* 2015;**105**:840. doi:10.7196/SAMJnew.8087

13 Ankomah SE, Fusheini A, Ballard C, *et al.* Patient-public engagement strategies for health system improvement in sub-Saharan Africa: a systematic scoping review. *BMC Health Serv Res* 2021;**21**:1047. doi:10.1186/s12913-021-07085-w

14 Pine H, Eisner ZJ, Delaney PG, *et al.* Prehospital Airway Management for Trauma Patients by First Responders in Six Sub-Saharan African Countries and Five Other Low- and Middle-Income Countries: A Scoping Review. *World J Surg* 2022;**46**:1396–407. doi:10.1007/s00268-022-06481-5

15 Mock CN, Quansah R, Addae-Mensah L, *et al.* The development of continuing education for trauma care in an African nation. *Injury* 2005;**36**:725–32. doi:10.1016/j.injury.2004.12.044

16 Bedard AF, Mata LV, Dymond C, Moreira F, Dixon J, Schauer SG, *et al.* A scoping review of worldwide studies evaluating the effects of prehospital time on trauma outcomes. *International Journal of Emergency Medicine* 2020 Vol. 13 Issue 1

17 Davies G and Chesters A, Transport of the trauma patient, *BJA: British Journal of Anaesthesia*, Volume 115, Issue 1, July 2015, Pages 33–37, https://doi.org/10.1093/bja/aev159

For peer review only

For peer review only - http://bmjopen.bmj.com/site/about/guidelines.xhtml

18 Caviglia M, Putoto G, Conti A, Tognon F, Jambai A, Vandy MJ, *et al.* Association between ambulance prehospital time and maternal and perinatal outcomes in Sierra Leone: a countrywide study. BMJ Glob Health 2021 Nov;6(11):e007315. doi: 10.1136/bmjgh-2021-007315

19 Ozawa S, Evans DR, Bessias S, et al. Prevalence and Estimated Economic Burden of Substandard and Falsified Medicines in Low- and Middle-Income Countries. JAMA Netw Open 2018;1:e181662. doi:10.1001/jamanetworkopen.2018.1662

20 Olaniran A, Briggs J, Pradhan A, *et al.* Stock-outs of essential medicines among community: health workers (CHWs) in low- and middle-income countries (LMICs): a systematic literature review of the extent, reasons, and consequences. *Hum Resour Health* 2022;**20**:58. doi:10.1186/s12960-022-00755-8

21 Stanford-Moore GB, Niyigaba G, Tuyishimire G, *et al.* Effect of Delay of Care for Patients with Craniomaxillofacial Trauma in Rwanda. *OTO Open* 2022;**6**:2473974X2210960. doi:10.1177/2473974X221096032

22 Rahman T, Gasbarro D, Alam K. Financial risk protection from out-of-pocket health spending in low- and middle-income countries: a scoping review of the literature. *Health Res Policy Syst* 2022;**20**:83. doi:10.1186/s12961-022-00886-3

23 Nolte E. Measuring the health of nations: analysis of mortality amenable to health care. *BMJ* 2003;**327**:1129–0. doi:10.1136/bmj.327.7424.1129

24 Mock CN, Gloyd S, Adjei S, *et al.* Economic consequences of injury and resulting family coping strategies in Ghana. *Accid Anal Prev* 2003;**35**:81–90. doi:10.1016/S0001-4575(01)00092-6

25 Niyigena A, Alayande B, Bikorimana L. *et al.* The true costs of cesarean delivery for patients in rural Rwanda: Accounting for post-discharge expenses in estimated health expenditures. *Int J Equity Health* **21**, 62 (2022). https://doi.org/10.1186/s12939-022-01664-x

26 Sapkota T, Houkes I, Bosma H. Vicious cycle of chronic disease and poverty: a qualitative study in present day Nepal. *Int Health* 2021;**13**:30–8. doi:10.1093/inthealth/ihaa016

27 ANANE, A (2021). DOCTOR-PATIENT COMMUNICATION IN THE CONSULTING ROOM: A STUDY OF TWO UNIVERSITY HEALTH FACILITIES IN GHANA. Afribary. Retrieved from: https://afribary.com/works/doctor-patient-communication-in-the-consulting-room-a-study-of-two-university-health-facilities-in-ghana.

28 Ifesanya A, Ifesanya J, Ogundele O. Orthopaedic surgical treatment delays at a tertiary hospital in sub Saharan Africa: Communication gaps and implications for clinical outcomes. *Nigerian Medical Journal* 2013;**54**:420. doi:10.4103/0300-1652.126301

For peer review only - http://bmjopen.bmj.com/site/about/guidelines.xhtml

29 Abang I, Asuquo J, Ngim N, *et al.* Reasons for patronage of traditional bone setters. *Nigerian Journal of Surgery* 2016;**22**:102. doi:10.4103/1117-6806.188993

30 Dickson DC, Christie SA, Chichom Mefire A, *et al.* A Qualitative Study of Transitions Between Health Care Settings After Injury in Cameroon. *Journal of Surgical Research* 2019;**244**:528–39. doi:10.1016/j.jss.2019.06.098

31 Garikapati V, Konadu-Yeboah D, Mengesha MG, *et al.* Systematic review of complications associated with treatment by traditional bone setters for musculoskeletal injury. *Trop Doct* 2022;:004947552211225. doi:10.1177/00494755221122543

32 Chu KM, Marco J, Owolabi EO, *et al.* Trauma trends during <scp>COVID</scp> -19 alcohol prohibition at a South African regional hospital. *Drug Alcohol Rev* 2022;**41**:13–9. doi:10.1111/dar.13310

33 Odland ML, Whitaker J, Nepogodiev D, *et al.* Identifying, Prioritizing and Visually Mapping Barriers to Injury Care in Rwanda: A Multi-disciplinary Stakeholder Exercise. *World J Surg* 2020;**44**:2903–18. doi:10.1007/s00268-020-05571-6

For peer review only

Equi-Trauma Collaborative:

Ciaran Kennedy[1], Agnieszka Ignatowicz[1], Maria Lisa Odland[1,2,3,4,5], Abdul-Malik Abdul-Latif[1,6], Antonio Belli[7,8], Anthony Howard[9,10], John Whitaker[1,11], Kathryn M. Chu[12,13], Karen Ferreira[12], Eyitayo O. Owolabi[12], Samukelisiwe Nyamathe[12], Stephen Tabiri[14,15,16], Bernard Ofori[14], Sheba Mary Pognaa Kunfah[14], Mustapha Yakubu[15,17], Abebe Bekele[18,19], Barnabas Alyande[20,21], Pascal Nzasabimana[22], Jean-Claude Byiringiro[22,23] and Justine Davies[1,12,24]

1. Institute of Applied Health Research, University of Birmingham, Birmingham, UK

2. Department of Obstetrics and Gynaecology, St. Olavs University Hospital, Trondheim, Norway

3. Malawi-Liverpool-Wellcome Trust Research Institute, Blantyre, Malawi

4. Department of Public Health and Nursing, NTNU, Norwegian University of Science and Technology, Trondheim, Norway

5. Institute of Life Course and Medical Sciences, University of Liverpool, Liverpool, United Kingdom

6. Volta Regional Health Directorate, Ghana Health Service, Accra, Greater Accra, Ghana

7. Institute of Inflammation and Ageing, University of Birmingham, Birmingham, UK

8. National Institute for Health Research Surgical Reconstruction and Microbiology Research Centre, Birmingham, UK

9. Leeds Institute of Rheumatic and Musculoskeletal Medicine, School of Medicine, University of Leeds, Leeds, UK

10. Nuffield Department of Orthopaedics, Rheumatology and Musculoskeletal Sciences, National Institute of Health Research (NIHR) Biomedical Centre, University of Oxford, Headington, Oxford, UK

11. King's Centre for Global Health and Health Partnerships, King's College London Faculty of Life Sciences and Medicine, London, UK

12. Centre for Global Surgery, Department of Global Health, Stellenbosch University, Cape Town, South Africa

13. Department of Surgery, University of Botswana, Gaborone, Botswana

14. Ghana HUB of NIHR Global Surgery, Tamale, Ghana

15. Department of Public Health, Tamale Teaching Hospital, Tamale, Ghana

16. Department of Surgery, Tamale Teaching Hospital, Tamale, Ghana

17. School of Medicine and Health Sciences, University for Development Studies, Tamale, Ghana

18. University of Global Health Equity, Kigali, Rwanda

19. Department of Surgery, Addis Ababa University, Addis Ababa, Ethiopia

20. Center for Equity in Global Surgery, University of Global Health Equity, Kigali, Rwanda

21. Program in Global Surgery and Social Change, Harvard Medical School, Boston, Massachusetts, USA

For peer review only

22. University of Rwanda, Kigali, Rwanda

23. Department of Surgery, University Teaching Hospital of Kigali, Kigali, Rwanda

24. Medical Research Council/Wits University Rural Public Health and Health Transitions Research Unit, Faculty of Health Sciences, School of Public Health, University of the Witwatersrand, Johannesburg, South Africa

For peer review only

Appendices

Appendix 1. The English version of the topic guide

Equitable access to quality trauma systems in Lower and Middle Income Countries. Assessing gaps and developing priorities

Service User In-Depth Interview Topic Guide Version 1. 25-01-2020 (note this is a discussion guide, the research assistants running the study will be fluent in English and local languages, and will translate discussion points, where needed, for participants)

First, I would like to thank you for attending this interview.

The recordings will be anonymous, and we don't want you to say your name at the start, but we have given each of you a pseudonym (made up name), and will go around the table in turn asking you to say your pseudonym, what age you are, the area in which you live.

This interview is to understand what would happen in <u>your community</u> if someone suffered an injury.

I would like to think particularly about four different aspects of what would happen, these aspects are <u>seeking care</u> (the time from an injury happens to taking action to get care), <u>reaching care</u> (the time until a facility or hospital that can treat the injury is reached), <u>receiving quality care</u> (getting good quality treatment for the injury at a facility or hospital), and <u>remaining in care</u> (for example, attending follow up at out-patients or going to rehabilitation).

I wish to know about your experiences and also your thoughts on whether others in your community have similar experiences.

I realise that you have had an injury in the last 6 months, could you tell us about what happened to cause the injury?

Where were you when the injury happened?

How about after your injury happened, what did you do?
<u>Prompts, if needed:</u>
- Did you think that you needed help?
- Was anyone there to help?
- Were you able to telephone for help?

Did you think that you needed to go and get treatment?
(If not, why not?)

Were there any things that stopped you trying to get treatment?
<u>Prompts, if needed</u>
- Knowledge of availability of healthcare?
- Trust in healthcare?
- Fear of costs?
- Availability of a phone?
- No time to go to healthcare?

Where did you first go to for treatment?
<u>Prompts, if needed:</u>
- Is that a clinic or a hospital?
- How far away was it?
- How long did it take to get there?
- If it took a long time to reach the care, why was that?

Was that the only place you went to, or did you need to (or were you referred to) another place to get care?

<u>Note to interviewer</u>: please explore how many places the person needed to go to in order to get definitive care

Were there any things in particular that delayed you getting to a hospital or clinic for care?
<u>Prompts, if needed:</u>
- Ambulance availability?
- Road conditions?
- Personal safety?
- Costs?

How about the care that you got in the facilities that you attended – do you think that the medical treatment was good?
<u>Prompts, if needed:</u>
- Were you seen quickly?
- Did the doctors and nurses seem competent?
- Do you think that they had all that they needed to treat your injury well?

How about how they treated you?
<u>Prompts, if needed:</u>
- Were you treated with respect?
- Did the staff explain things to you?

How about costs of care?
<u>Prompts, if needed:</u>
- Did you have to pay for care?
- Did you have to borrow or sell anything to pay for care?

Were there any things in particular that stopped you getting the right treatment in the hospital?
<u>Prompts if needed:</u>
- Family commitments meant had to shorten stay?
- Costs of care?
- Too far for relatives to travel to visit?

After your injury was treated, were you asked to go back to see healthcare workers about your injury? For example, doctors in a clinical appointment, or physiotherapists for rehabilitation?

If so, how far did you have to travel to get to these appointments?

Did you manage to keep all of the appointments?

Were there any things in particular that you felt limited your ability to keep all these appointments?
<u>Prompts, if needed:</u>
- Travel time?
- Costs?
- Other commitments?
- Thought there was no need?

Are you now back to your full fitness, do you think?

Do you think anything could have been done better?

Now that's the end of this discussion, thank-you for telling us about your experiences.
Now, we would be grateful if we could ask you some other questions about your time in the hospital or clinic. It will take another ten minutes.

For peer review only

Note to interviewer ask I-PAHC and/or O-PAHC questionnaire depending on whether the participant was admitted to hospital or was seen in a clinic, or both.

Does you have any questions for me?
Thank-you

For peer review only

Appendix 2. Barriers to good patient experience of trauma care, in descending order of frequency.

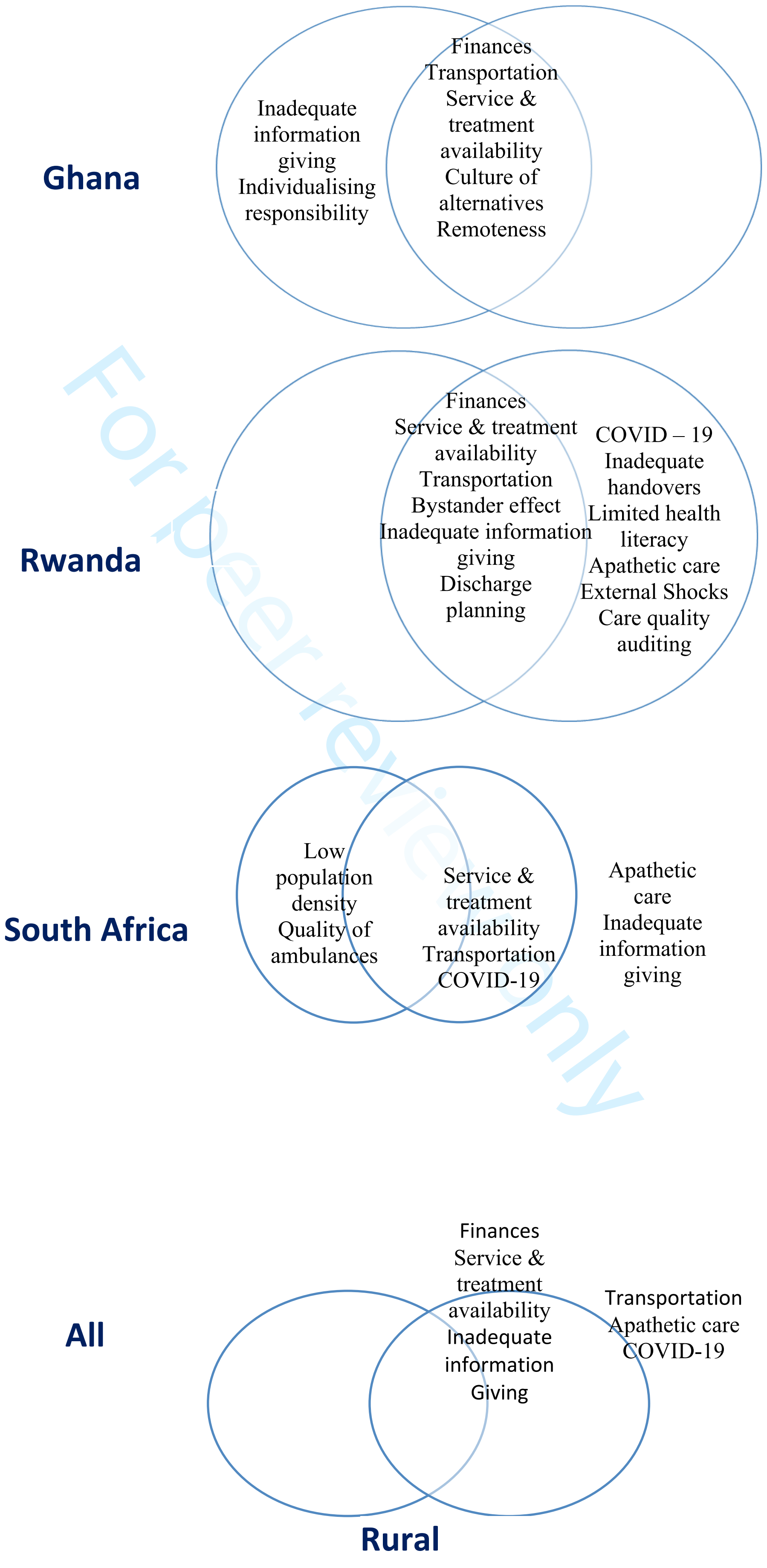

Appendix 3. Facilitators to good patient experience of trauma care, in descending order of frequency

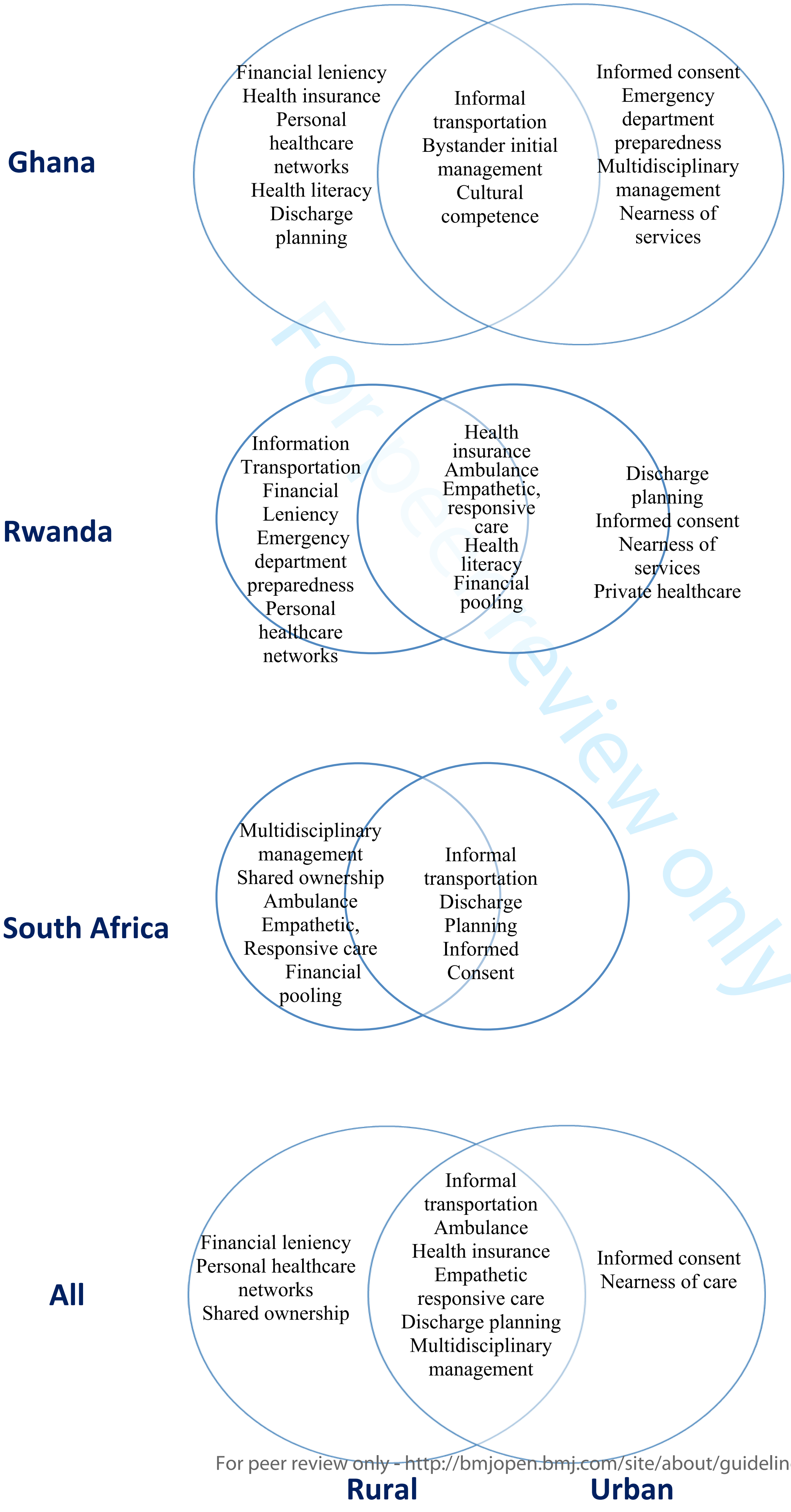

For peer review only

Appendix 4: Barriers to a good patient experience of trauma care in Ghana

	Ghana			
Barriers	Rural	Urban	Rural - Urban	Total
Financial Scarcity and inadequate healthcare coverage	10	9	1	19
Service and treatment availability	8	8	0	16
Transportation Barriers	7	8	-1	15
Cultural Value of alternative services	7	5	2	12
Inadequate Information Giving	7	3	4	10
Limited Health Literacy	2	8	-6	10
Apathetic Care	2	6	-4	8
Low Population Density	2	1	1	3
Narratives of Self-blame and Individualising Responsibility	2	0	2	2
Absence of care quality assessment	0	1	-1	1
COVID-19	0	1	-1	1
Absence of occupational health assessment	0	0	0	0
Bystander Effect	0	0	0	0
Care responsibilities	0	0	0	0
Clinician-patient power inequality	0	0	0	0
Delayed presented due to perceived low severity	0	0	0	0
Diagnostic uncertainty	0	0	0	0
Ethnic inequities in health system provision	0	0	0	0
Fear of surgery	0	0	0	0
Hospital catchment area system	0	0	0	0
Inability to self-care / loss of autonomy	0	0	0	0
Inadequate administration of Follow-Ups	0	0	0	0
Inadequate analgesia	0	0	0	0
Inadequate Discharge Planning	0	0	0	0
Inadequate Handovers	0	0	0	0
Language Barriers	0	0	0	0
Limited Specialist Training	0	0	0	0
Loss to follow up	0	0	0	0
Obstruction	0	0	0	0
Poor communication with family	0	0	0	0
Quality of ambulances	0	0	0	0
suboptimal clinical management	0	0	0	0
Unmet psychological needs	0	0	0	0
Unresponsiveness to formal complaints	0	0	0	0

For peer review only

Appendix 5: Barriers to a good patient experience of trauma care in Rwanda

	Rwanda			
Barriers	Rural	Urban	Rural - Urban	Total
Financial Scarcity and inadequate healthcare coverage	9	8	1	17
Service and treatment availability	7	9	-2	16
Transportation Barriers	7	8	-1	15
COVID-19	3	8	-5	11
Apathetic Care	5	3	2	8
Inadequate Discharge Planning	4	3	1	7
Bystander Effect	3	3	0	6
Inadequate Information Giving	3	2	1	5
Inadequate administration of Follow-Ups	2	1	1	3
Limited Health Literacy	3	0	3	3
Inadequate Handovers	0	2	-2	2
Absence of occupational health assessment	2	0	2	2
Obstruction	0	1	-1	1
Language Barriers	1	0	1	1
Limited Specialist Training	1	0	1	1
Diagnostic uncertainty	1	0	1	1
Clinician-patient power inequality	1	0	1	1
Inability to self-care / loss of autonomy	1	0	1	1
Narratives of Self-blame and Individualising Responsibility	1	0	1	1
Quality of ambulances	1	0	1	1
Inadequate analgesia	1	0	1	1
Cultural Value of alternative services	1	0	1	1
Hospital catchment area system	0	0	0	0
Ethnic inequities in health system provision	0	0	0	0
Care responsibilities	0	0	0	0
Unmet psychological needs	0	0	0	0
Fear of surgery	0	0	0	0
Loss to follow up	0	0	0	0
Unresponsiveness to formal complaints	0	0	0	0
Poor communication with family	0	0	0	0
Absence of care quality assessment	0	0	0	0
suboptimal clinical management	0	0	0	0
Delayed presented due to perceived low severity	0	0	0	0
Low Population Density	0	0	0	0

For peer review only

Appendix 6: Barriers to a good patient experience of trauma care in South Africa

	South Africa			
Barriers	Rural	Urban	Rural - Urban	Total
Apathetic Care	4	9	-5	13
Inadequate Information Giving	4	8	-4	12
Service and treatment availability	4	8	-4	12
Transportation Barriers	4	4	0	8
COVID-19	4	3	1	7
Financial Scarcity and inadequate healthcare coverage	3	3	0	6
suboptimal clinical management	1	2	-1	3
Delayed presented due to perceived low severity	2	1	1	3
Inadequate analgesia	1	2	-1	3
Unresponsiveness to formal complaints	0	2	-2	2
Poor communication with family	1	1	0	2
Absence of care quality assessment	0	2	-2	2
Inability to self-care / loss of autonomy	1	1	0	2
Quality of ambulances	2	0	2	2
Low Population Density	2	0	2	2
Inadequate Discharge Planning	0	2	-2	2
Hospital catchment area system	0	1	-1	1
Ethnic inequities in health system provision	0	1	-1	1
Care responsibilities	0	1	-1	1
Unmet psychological needs	0	1	-1	1
Fear of surgery	0	1	-1	1
Loss to follow up	0	1	-1	1
Inadequate administration of Follow-Ups	0	1	-1	1
Obstruction	0	0	0	0
Language Barriers	0	0	0	0
Limited Specialist Training	0	0	0	0
Diagnostic uncertainty	0	0	0	0
Clinician-patient power inequality	0	0	0	0
Inadequate Handovers	0	0	0	0
Absence of occupational health assessment	0	0	0	0
Narratives of Self-blame and Individualising Responsibility	0	0	0	0
Bystander Effect	0	0	0	0
Limited Health Literacy	0	0	0	0
Cultural Value of alternative services	0	0	0	0

For peer review only

Appendix 7: Facilitators to a good patient experience of trauma care in Ghana

	Ghana			
Facilitators	Rural	Urban	Rural - Urban	Total
Informal transportation	6	8	-2	14
Bystander Initial Management	2	3	-1	5
Financial Leniency / Charity	4	0	4	4
Health Insurance	4	0	4	4
Information giving & informed consent	0	4	-4	4
Personal Healthcare Networks	3	0	3	3
Health Literacy	3	0	3	3
Ambulance	2	1	1	3
Cultural Competence	0	2	-2	2
Nearness of Care	0	2	-2	2
Emergency Department Preparedness	0	2	-2	2
Multidisciplinary Management	0	2	-2	2
Community or Familial Financial Pooling	0	1	-1	1
Discharge Planning	1	0	1	1
Preferential Treatment	0	0	0	0
Police-facilitated transport	0	0	0	0
Communications Network	0	0	0	0
Familial Rehabilitative Support	0	0	0	0
Patient autonomy	0	0	0	0
NGO Support	0	0	0	0
Provision of mobility aids	0	0	0	0
Requesting Timely Care	0	0	0	0
Private Healthcare	0	0	0	0
Shared ownership of management plan	0	0	0	0
Empathetic Responsive care	0	0	0	0

For peer review only

Appendix 8: Facilitators to a good patient experience of trauma care in Rwanda

	Rwanda			
Facilitators	Rural	Urban	Rural - Urban	Total
Health Insurance	8	9	-1	17
Ambulance	6	8	-2	14
Information giving & informed consent	4	8	-4	12
Informal transportation	9	3	6	12
Empathetic Responsive care	4	6	-2	10
Health Literacy	4	5	-1	9
Community or Familial Financial Pooling	3	4	-1	7
Financial Leniency / Charity	5	2	3	7
Bystander Initial Management	2	4	-2	6
Discharge Planning	0	5	-5	5
NGO Support	2	1	1	3
Communications Network	1	1	0	2
Nearness of Care	0	2	-2	2
Private Healthcare	0	2	-2	2
Multidisciplinary Management	1	1	0	2
Preferential Treatment	1	0	1	1
Patient autonomy	0	1	-1	1
Cultural Competence	0	1	-1	1
Emergency Department Preparedness	1	0	1	1
Requesting Timely Care	1	0	1	1
Personal Healthcare Networks	1	0	1	1
Police-facilitated transport	0	0	0	0
Familial Rehabilitative Support	0	0	0	0
Provision of mobility aids	0	0	0	0
Shared ownership of management plan	0	0	0	0

For peer review only

Appendix 9: Facilitators to a good patient experience of trauma care in South Africa

	South Africa			
Facilitators	Rural	Urban	Rural - Urban	Total
Informal transportation	7	9	-2	16
Multidisciplinary Management	8	2	6	10
Discharge Planning	5	4	1	9
Empathetic Responsive care	7	1	6	8
Information giving & informed consent	4	4	0	8
Ambulance	6	1	5	7
Shared ownership of management plan	5	0	5	5
Provision of mobility aids	3	0	3	3
Community or Familial Financial Pooling	3	0	3	3
Requesting Timely Care	2	1	1	3
Private Healthcare	2	1	1	3
Familial Rehabilitative Support	1	1	0	2
Bystander Initial Management	1	1	0	2
Police-facilitated transport	1	0	1	1
Patient autonomy	1	0	1	1
Emergency Department Preparedness	1	0	1	1
Personal Healthcare Networks	1	0	1	1
Financial Leniency / Charity	1	0	1	1
Health Insurance	1	0	1	1
Preferential Treatment	0	0	0	0
Communications Network	0	0	0	0
Cultural Competence	0	0	0	0
NGO Support	0	0	0	0
Nearness of Care	0	0	0	0
Health Literacy	0	0	0	0

For peer review only

Appendix 10. ***Thematic corroboration of results with the findings of the prior mixed-methods study in Ghana, Rwanda and South Africa.***

Barriers	Barriers (Odland et al.)	Facilitators	Facilitators (Odland et al.)
Absence of care quality assessment	Present	Ambulance	Present
Absence of occupational health assessment		Bystander Initial Management	Present
Apathetic Care		Communications Network	
Bystander Effect	Present	Community or Familial Financial Pooling	
Care responsibilities		Cultural Competence	Present
Clinician-patient power inequality	Present	Discharge Planning	Present
COVID-19	Present	Emergency Department Preparedness	Present
Cultural Value of alternative services	Present	Empathetic Responsive care	Present
Delayed presented due to perceived low severity		Familial Rehabilitative Support	
Diagnostic uncertainty		Financial Leniency / Charity	
Inequities in health system provision	Present	Health Literacy	Present
Fear of surgery		Health Insurance	Present
Individual financial scarcity and inadequate health insurane coverage	Present	Informal transportation	
Hospital catchment area system	Present	Information giving and informed consent	Present
Inability to self-care / loss of autonomy		Multidiscliplinary Management	Present
Inadequate administration of Follow-Ups	Present	Nearness of Care	Present
Inadequate analgesia	Present	NGO Support	
Inadequate Discharge Planning	Present	Patient autonomy	Present
Inadequate Handovers		Personal Healthcare Networks	
Inadequate Information Giving	Present	Police-facilitated transport	
Language Barriers		Preferential Treatment	
Service and treatment availability	Present	Private Healthcare	
Health literacy	Present	Provision of mobility aids	
Limited Specialist Training		Requesting Timely Care	
Low Population Density	Present	Shared ownership of management plan	Present
Narratives of Self-blame and Individualising Responsibility			Coinciding themes (%)
Obstruction			52
Poor communication with family			
Quality of ambulances	Present		
Suboptimal clinical management			
Transportation Barriers	Present		
Unmet psychological needs	Present		
Unresponsiveness to formal complaints	Present		
Loss to Follow Up			
	Coinciding themes (%)		
	60.60606061		

For peer review only

COREQ (COnsolidated criteria for REporting Qualitative research) Checklist

A checklist of items that should be included in reports of qualitative research. You must report the page number in your manuscript where you consider each of the items listed in this checklist. If you have not included this information, either revise your manuscript accordingly before submitting or note N/A.

Topic	Item No.	Guide Questions/Description	Reported on Page No.
**Domain 1: Research team and reflexivity**			
Personal characteristics			
Interviewer/facilitator	1	Which author/s conducted the interview or focus group?	
Credentials	2	What were the researcher's credentials? E.g. PhD, MD	
Occupation	3	What was their occupation at the time of the study?	
Gender	4	Was the researcher male or female?	
Experience and training	5	What experience or training did the researcher have?	
Relationship with participants			
Relationship established	6	Was a relationship established prior to study commencement?	
Participant knowledge of the interviewer	7	What did the participants know about the researcher? e.g. personal goals, reasons for doing the research	
Interviewer characteristics	8	What characteristics were reported about the inter viewer/facilitator? e.g. Bias, assumptions, reasons and interests in the research topic	
**Domain 2: Study design**			
Theoretical framework			
Methodological orientation and Theory	9	What methodological orientation was stated to underpin the study? e.g. grounded theory, discourse analysis, ethnography, phenomenology, content analysis	
Participant selection			
Sampling	10	How were participants selected? e.g. purposive, convenience, consecutive, snowball	
Method of approach	11	How were participants approached? e.g. face-to-face, telephone, mail, email	
Sample size	12	How many participants were in the study?	
Non-participation	13	How many people refused to participate or dropped out? Reasons?	
Setting			
Setting of data collection	14	Where was the data collected? e.g. home, clinic, workplace	
Presence of non-participants	15	Was anyone else present besides the participants and researchers?	
Description of sample	16	What are the important characteristics of the sample? e.g. demographic data, date	
Data collection			
Interview guide	17	Were questions, prompts, guides provided by the authors? Was it pilot tested?	
Repeat interviews	18	Were repeat inter views carried out? If yes, how many?	
Audio/visual recording	19	Did the research use audio or visual recording to collect the data?	
Field notes	20	Were field notes made during and/or after the inter view or focus group?	
Duration	21	What was the duration of the inter views or focus group?	
Data saturation	22	Was data saturation discussed?	
Transcripts returned	23	Were transcripts returned to participants for comment and/or	

For peer review only

Topic	Item No.	Guide Questions/Description	Reported on Page No.
		correction?	
**Domain 3: analysis and findings**			
Data analysis			
Number of data coders	24	How many data coders coded the data?	
Description of the coding tree	25	Did authors provide a description of the coding tree?	
Derivation of themes	26	Were themes identified in advance or derived from the data?	
Software	27	What software, if applicable, was used to manage the data?	
Participant checking	28	Did participants provide feedback on the findings?	
Reporting			
Quotations presented	29	Were participant quotations presented to illustrate the themes/findings? Was each quotation identified? e.g. participant number	
Data and findings consistent	30	Was there consistency between the data presented and the findings?	
Clarity of major themes	31	Were major themes clearly presented in the findings?	
Clarity of minor themes	32	Is there a description of diverse cases or discussion of minor themes?	

Developed from: Tong A, Sainsbury P, Craig J. Consolidated criteria for reporting qualitative research (COREQ): a 32-item checklist for interviews and focus groups. *International Journal for Quality in Health Care*. 2007. Volume 19, Number 6: pp. 349 – 357

Once you have completed this checklist, please save a copy and upload it as part of your submission. DO NOT include this checklist as part of the main manuscript document. It must be uploaded as a separate file.

For peer review only

BMJ Open

Commonalities and differences in injured patient experiences of accessing and receiving quality injury care: a qualitative study in three sub-Saharan African countries

Journal:	*BMJ Open*
Manuscript ID	bmjopen-2023-082098.R1
Article Type:	Original research
Date Submitted by the Author:	13-May-2024
Complete List of Authors:	The Equi-Trauma Collaborative , The Equi-Trauma Collaborative ; University of Birmingham College of Medical and Dental Sciences<br>Ignatowicz, Agnieszka ; University of Birmingham Institute of Applied Health Research
**Primary Subject Heading**:	Global health
Secondary Subject Heading:	Qualitative research
Keywords:	Quality in health care < HEALTH SERVICES ADMINISTRATION & MANAGEMENT, Patient-Centered Care, QUALITATIVE RESEARCH

SCHOLARONE™
Manuscripts

I, the Submitting Author has the right to grant and does grant on behalf of all authors of the Work (as defined in the below author licence), an exclusive licence and/or a non-exclusive licence for contributions from authors who are: i) UK Crown employees; ii) where BMJ has agreed a CC-BY licence shall apply, and/or iii) in accordance with the terms applicable for US Federal Government officers or employees acting as part of their official duties; on a worldwide, perpetual, irrevocable, royalty-free basis to BMJ Publishing Group Ltd ("BMJ") its licensees and where the relevant Journal is co-owned by BMJ to the co-owners of the Journal, to publish the Work in this journal and any other BMJ products and to exploit all rights, as set out in our licence.

The Submitting Author accepts and understands that any supply made under these terms is made by BMJ to the Submitting Author unless you are acting as an employee on behalf of your employer or a postgraduate student of an affiliated institution which is paying any applicable article publishing charge ("APC") for Open Access articles. Where the Submitting Author wishes to make the Work available on an Open Access basis (and intends to pay the relevant APC), the terms of reuse of such Open Access shall be governed by a Creative Commons licence – details of these licences and which Creative Commons licence will apply to this Work are set out in our licence referred to above.

Other than as permitted in any relevant BMJ Author's Self Archiving Policies, I confirm this Work has not been accepted for publication elsewhere, is not being considered for publication elsewhere and does not duplicate material already published. I confirm all authors consent to publication of this Work and authorise the granting of this licence.

For peer review only

Commonalities and differences in injured patient experiences of accessing and receiving quality injury care : a qualitative study in three sub-Saharan African countries

The Equi-Trauma Collaborative

(the full list of authors, their contributions, and affiliations are at the end of the manuscript)

Corresponding authors:

Dr Agnieszka Ignatowicz and Dr Ciaran Kennedy, Institute of Applied Health Research, University of Birmingham, Birmingham, B15 2TT, UK. E-mail: a.m.ignatowicz@bham.ac.uk

Acknowledgements:

We would like to acknowledge the work of our co-author Mr Abul Malik Abul-Latif whose tireless work enabled the completion of this study. Abdul passed away in September 2023.

Abstract

Objectives: To understand commonalities and differences in injured patient experiences of accessing and receiving quality injury care across three lower- and middle-income countries.

Design: A qualitative interview study. The interviews were audiorecorded, transcribed and thematically analysed.

Setting: Urban and rural settings in Ghana, South Africa, and Rwanda.

Participants: 59 patients with musculoskeletal injuries.

Results: We found five common barriers and six common facilitators to injured patient experiences of accessing and receiving high-quality injury care. The barriers encompassed issues such as service and treatment availability, transportation challenges, apathetic care, individual financial scarcity and inadequate health insurance coverage, alongside low health literacy and information provision. Facilitators included effective information giving and informed consent practices, access to health insurance, improved health literacy, empathetic and responsive care, comprehensive multidisciplinary management and discharge planning, as well as both informal and formal transportation options including ambulance services. These barriers and facilitators were prevalent and shared across at least two countries but demonstrated inter-country and intra-country (between urbanity and rurality) variation in thematic frequency.

Conclusion: There are universal factors influencing patient experiences of accessing and receiving care, independent of the context or healthcare system. It is important to recognize and understand

For peer review only - http://bmjopen.bmj.com/site/about/guidelines.xhtml

these barriers and facilitators to inform policy decisions and develop transferable interventions aimed at enhancing the quality of injury care in sub-Saharan African nations.

Strengths and limitations of this study

- The data collection for this study was conducted during periods of national COVID-19 lockdowns in Ghana, South Africa and Rwanda, which may have affected the quality and depth of the data.
- Only patients with musculoskeletal injuries were included in the study sample.
- We employed multiple analysts and validated our findings by triangulating them with existing literature to increase the credibility of our results.

Introduction

Approximately 90% of injury-related mortality occurs in low- and middle-income countries (LMICs) [1]. Across the African continent, the past two decades have witnessed a near 50% rise in healthy life-years lost from road traffic incidents [1]. This increased prevalence of injury has significant implications for health system capacity and finances [2]. Whilst preventative measures are urgently needed to reduce injury prevalence, access to quality healthcare for injured individuals is also essential to save lives and prevent disability [3, 4]. Unfortunately, the past 25 years have seen a critical underfunding of injury care, compared to other global health concerns [5]. The establishment of a *sustainable development goal* target (3.6) to halve global deaths and injuries from road traffic accidents by 2030, may help to redress this balance [6].

Ensuring equitable access to high-quality care following an injury is essential step in improving health outcomes in LMICs [7, 8]. Adopting a comprehensive approach that tends to the needs of injured individuals from the moment of injury until optimal rehabilitation can improve both mortality and morbidity rates [4]. However, knowledge of barriers to accessing care after injury is limited [9] and has mostly been studied at the healthcare facility level; studies seldomly look across the whole patient pathway, from point of injury to discharge from care [7]. Those studies that have explored the whole pathway have identified multiple barriers, with the majority (around 60%) focussing on barriers to receiving quality care [8].Identifying how to increase equitable access to injury care will require a whole health system approach to injury research, not one solely focussed on the capacity for patients to receive acute care [7-9].

For peer review only - http://bmjopen.bmj.com/site/about/guidelines.xhtml

Exploring patient experiences of injury and injury care along the whole care pathway can contribute towards a systems-level understanding of injury care. Utilising qualitative methodology enables movement beyond simply delineating barriers to in access to services, to assessing the *acceptability* of services and subjective facilitators of quality injury care [8]. It also has the potential to elicit contextually dependent solutions to access barriers. Exploring these perspectives across multiple LMICs and foregrounding their commonalities may afford opportunities to improve health across multiple nations through transferable interventions. Therefore, our aim in this paper was to understand commonalities and differences in injured patient experiences of accessing and receiving quality injury care across three lower- and middle-income countries.

Methods

This analysis used qualitative data collected from the *Equi-Trauma* study conducted in Ghana, South Africa, and Rwanda. It is reported according to the COREQ (Consolidated criteria for reporting qualitative research) checklist [10].

Participants and sampling strategy

Methods for the main study have been published elsewhere [8]. For the main study, in each country we aimed to recruit 10 participants in both the urban and rural areas to ensure a relatively equal number of patients. Convenience sampling was employed to select persons in communities who had been injured and accessed – or attempted to access – care for an injury which occurred in the previous six months. Where possible, purposive sampling was done to enable representation of individuals across ages, sexes, and injury types. Hospital lists and contact with community leaders were used to identify participants who had or had not presented to clinics or hospitals for their injuries. When injured patients were not able to participate (for example, after death or disability), friends or relatives present during the injury were invited to participate. For this paper, we only included participants that had a musculoskeletal injury as this was the main mechanisms of injury across the four study countries.

Data collection process

Face-to-face in-depth interviews lasting up to an hour were conducted between January and October 2021 in urban and rural settings in all three countries. Interviews were conducted in the participants' preferred language and at locations suitable for participants, including hospitals, clinics, or patients' homes. Participants were interviewed by a male and female surgeon in Ghana (SW and MY), a male non-clinical researcher in Rwanda (PN), and two female junior doctors in South Africa (KF and SN), all native to the country in which data were collected. . If interviews were not conducted in English,

For peer review only - http://bmjopen.bmj.com/site/about/guidelines.xhtml

translation was conducted by in-country researchers (KF, MY, PN, SN and SW) and checked by in-country investigators (KC, JCB, ST).

Data collection tools

All interviewers were trained by a senior member of the research team (AI) to ensure standardisation of data collection methods. The English version of the topic guide, before adaptation to local contexts, is attached (append. 1). This was developed to enable a rich understanding of patients' experiences of injury care along the whole care pathway from initial injury to rehabilitation. The topic guide was based on the authors' experiences and knowledge of the literature, permitting interviewer discretion to explore themes that emerged in specific interviews. It was piloted, and interviewers received feedback from a qualitative methods expert (AI) on their interviewing technique and field notes after two preliminary interviews. In all three countries, data saturation was reached towards the last interviews, reflecting the complexity of contexts and richness of the data.

Analytical approach

Interviews were audio-recorded and then transcribed verbatim. Computer-assisted thematic content analysis was conducted using *QSR NVivo* [11]. Codes were generated inductively, focussing on perceived barriers and facilitators to accessing quality injury care. A sample of interview transcripts across three study countries was read to identify the initial set of codes by two co-authors (AI and CK) through serial discussions. This generated an initial coding framework that was discussed in an analysis meeting between members of the research team (AI, CK and JD) and then used to code all remaining interview transcripts. Codes were gradually built into broader categories, with final themes being selected through comparison across transcripts and through discussion amongst co-authors, resolving discrepancies. Barriers and facilitators were coded as such dependent on how the participant reported them. Hence, themes could have been reported both as a barrier and a facilitator, depending on if the participants reported them negatively or positively. Some themes with perceived substantial thematic overlap were paired as a result of the further discussions between the two main researchers (AI & CK). Paired themes were reported together.

Barriers and facilitators that were shared across at least two countries were identified, as well as discordant themes that were prevalent in only one country. The patient-reported barriers and facilitators to good experience of trauma care for each country are available in appendices (append.2-3). We then corroborated our results with the findings of our prior mixed-methods study in Ghana, Rwanda and South Africa [8].

For peer review only - http://bmjopen.bmj.com/site/about/guidelines.xhtml

Description of sample

A total of 60 in-depth interviews were conducted across Ghana, Rwanda, and South Africa, evenly segmented by urban versus rural areas, as part of the main study. Ghanaian interviews were held in the Northern regional capital of Tamale and rural Yendi district (three female and 17 male injured individuals). Rwandan interviews were held in the capital city of Kigali and rural Burera district of the Northern Province (five female and 15 male injured individuals). South African interviews were held in the urban township Khayelitsha and across the rural Western Cape (four female and 16 male injured individuals). Most of the persons interviewed in the main study had experienced musculoskeletal injuries and were eligible for inclusion into this study. For this paper, we only included participants that had a musculoskeletal injury as this was the main mechanisms of injury across the four study countries.

Ethical approval

Data collection was approved by: the Ghana Health Service Ethics Review Committee (GHS-ERC005/02/20); The Stellenbosch University Health Research Ethics Committee for South Africa (N20/01/010) and the Internal Research Board of the College of Medicine and Health Sciences of the University of Rwanda for Rwanda (No 050/CMHS IRB/2020). Additional approval was obtained from the Western Cape Department of Health (WC_202006_022) in South Africa, and from the respective hospitals before visiting the facilities. The overall study was approved by the University of Birmingham Research Ethics Committee, UK (ERN_20-00880). Funding was provided by the National Institute of Health Research, NIHR, award number 130036.

Patient and public involvement

Patients and/or the public were not involved in the design, reporting, or dissemination plans of this research.

Authors' reflexivity statement

The Equi-Trauma Collaborative was a research team comprising academics, researchers and clinicians based in the UK, Rwanda, Ghana and South Africa. The team members had diverse cultural backgrounds. CK, AI, JD, MLO, ABell, AH and JW represented high-income country researchers. The knowledge of lead investigators (KC, JC, ST) and involvement of local researchers in each study country have been crucial to the development and success of this project. All research staff who engaged in data collection are acknowledged as authors; specific roles of authors are outlined in this methods section and acknowledgments. The NIHR grant that funded this project was used to support the local

For peer review only - http://bmjopen.bmj.com/site/about/guidelines.xhtml

research teams to undertake data collection, purchase qualitative analytical software, and disseminate the findings. Multiple virtual meetings were held with all research partners to train staff to undertake data collection, analyse the data and write up the results. There are three other manuscripts published as a result of this research, two of which are first authored by the early career researchers from Rwanda and South Africa [12, 13]. The manuscript from Ghana is currently in preparation.

The findings from this research formed the basis of an application for a larger grant that has been successful in securing the funding (Equi-injury: NIHR Global Health Group on Equitable Access to Quality Health Care for Injured People in Four Low or Middle Income Countries). The project is co-led by the PIs based in the UK and South Africa. With small changes to the team in the UK and addition of another partner country (Pakistan), we have formed the Equi-injury Group. There is a large capacity building component attached to this project, which focuses on building sustainable partnerships and South-South learning.

Results

All individuals approached agreed to participate in the study with an attrition rate of 0%. Several themes were paired together due to perceived substantial thematic overlap between them, a decision reached through discussions between researchers (AI & CK) during the analysis and interpretation of findings stage. These were: '*individual financial scarcity*' and '*inadequate health insurance coverage'*; '*empathetic care*' and '*responsive care'*, and '*information giving*' and '*informed consent*' . Some themes were aggregated (summarized and reported together to provide an overall view because of the need to keep the Results section condensed) and included: *'health literacy' and 'information giving', 'informal transportation' and 'ambulance transportation', 'multidisciplinary management' and 'discharge planning'.*

A total of 34 unique barriers and 25 unique facilitators to accessing perceived quality injury care were identified across the three countries (Tables 1 and 2). In Ghana there were 11 barriers and 14 facilitators; in Rwanda, 22 barriers and 21 facilitators; and in South Africa 23 barriers and 19 facilitators. Barriers and facilitators by rural or urban setting per country are presented in appendices (append.4-9). Direct quotations are shown for the most prevalent barriers common to each country and the most prevalent facilitators common to each country.

For peer review only - http://bmjopen.bmj.com/site/about/guidelines.xhtml

Table 1: Perceived barriers to a good patient experience of trauma care across Ghana, Rwanda & South Africa. The number refers to the number of times the barrier was reported. The most prevalent barriers are in red.

Barriers	All Countries Total number	Rural	Urban
Service and treatment availability	44	19	25
Financial scarcity and inadequate healthcare coverage	42	22	20
Transportation Barriers	38	18	20
Apathetic Care	29	11	18
Inadequate Information Giving	27	14	13
COVID-19	19	7	12
Cultural Value of alternative services	13	8	5
Limited Health Literacy	13	5	8
Inadequate Discharge Planning	9	4	5
Bystander Effect	6	3	3
Low Population Density	5	4	1
Inadequate administration of Follow-Ups	4	2	2
Inadequate analgesia	4	2	2
Quality of ambulances	3	3	0
Narratives of Self-blame and Individualising Responsibility	3	3	0
Inability to self-care / loss of autonomy	3	2	1
Delayed presented due to perceived low severity	3	2	1
Suboptimal clinical management	3	1	2
Absence of care quality assessment	3	0	3
Poor communication with family	2	1	1
Absence of occupational health assessment	2	2	0
Unresponsiveness to formal complaints	2	0	2
Inadequate Handovers	2	0	2
Loss to follow up	1	0	1
Limited Specialist Training	1	1	0
Diagnostic uncertainty	1	1	0
Language Barriers	1	1	0
Clinician-patient power inequality	1	1	0
Hospital catchment area system	1	0	1
Care responsibilities	1	0	1
Unmet psychological needs	1	0	1
Fear of surgery	1	0	1
Obstruction	1	0	1
Ethnic inequities in health system provision	1	0	1

For peer review

Table 2: Perceived facilitators to a good patient experience of trauma care across Ghana, Rwanda & South Africa. The number refers to the number of times the barrier was reported. The most prevalent barriers are in red.

**Facilitators**	**All Countries**		
	**Total number**	**Rural**	**Urban**
**Informal transportation**	42	22	20
**Information giving & informed consent**	24	8	16
**Ambulance**	24	14	10
**Health Insurance**	22	13	9
**Empathetic Responsive care**	18	11	7
**Discharge Planning**	15	6	9
**Multidisciplinary Management**	14	9	5
**Bystander Initial Management**	13	5	8
**Health Literacy**	12	7	5
**Financial Leniency / Charity**	12	10	2
**Community or Familial Financial Pooling**	11	6	5
**Private Healthcare**	5	2	3
**Personal Healthcare Networks**	5	5	0
**Shared ownership of management plan**	5	5	0
**Nearness of Care**	4	0	4
**Emergency Department Preparedness**	4	2	2
**Requesting Timely Care**	4	3	1
**Cultural Competence**	3	0	3
**NGO Support**	3	2	1
**Provision of mobility aids**	3	3	0
**Communications Network**	2	1	1
**Familial Rehabilitative Support**	2	1	1
**Patient autonomy**	2	1	1
**Preferential Treatment**	1	1	0
**Police-facilitated transport**	1	1	0

1. Common Barriers

Five barriers were shared and prevalent across at least two countries (Table 1). Less prevalent barriers are reported with their thematic frequencies in appendices (append.4-6)

1.1 Service and treatment availability

This broad theme was coded 16 times in Ghanaian interviews, 16 times in Rwandan interviews and 12 times in South African interviews.

For peer review only

Some respondents noted an absence of adequate care in ambulances. On arrival at hospital, a lack of acute care beds and the lack of capacity to see patients in a timely manner was noted. Slow initial reviews were reportedly contributed to by inadequate staffing levels (particularly during religious and cultural festivals) and escalating patients to a more specialised hospital before providing initial management. The unavailability of essential services (such as blood transfusion and medical imaging) at certain hospitals in Ghana sometimes necessitated referrals. Other services in Ghana were reportedly not offered, including physiotherapy and occupational therapy. Respondents in Rwanda noted that some hospitals had low medicinal stock and placed the onus on patients to purchase their in-patient medications from pharmacies - sometimes situated at a distance from the hospital. Respondents in South Africa identified the lack of provision of weekend theatre lists and the tendency to postpone operations after a full day of fasting.

1.2 Transportation barriers

This broad theme was coded 15 times in interviews from Ghana, 15 times in interviews from Rwanda and eight times in interviews from South Africa.

Many respondents relied on the whims of passing vehicles to reach definitive care. Some respondents in rural Ghana noted this opportunistic transport seldom arrived due to the low population density, whilst some respondents in Rwanda stated that it was not feasible to use opportunistic motorcycles due to the degree of injury. Transportation was sometimes further delayed for Rwandan respondents due to the requirements of thorough police investigations.

There was consensus amongst most urban and rural Ghanaian respondents that there were no ambulances available for use, whilst Rwandan and South African respondents noted ambulances were often inexplicably delayed. Respondents in urban South Africa reported their area was not serviced by ambulances due to impenetrably dense shacks and/or ambulance staffs' fears of robbery. Rural South African respondents noted that ambulances operated like a taxi services, collecting multiple patients from the scene of each injury before reaching definitive care.

Many patients were referred to multiple facilities from low to highly specialised centres for treatment, until an appropriate hospital for their injury was reached – each escalation necessitating further transport. Rwandan respondents noted the scarcity of ambulances for inter-hospital transfers. Ghanaian respondents noted that the crowdedness of public transport (notably buses) led to some

For peer review only - http://bmjopen.bmj.com/site/about/guidelines.xhtml

individuals not seeking follow-up care. Some Rwandan interviewees reported reaching follow-up by foot due to a lack of public transport, exacerbated by COVID-19-associated road and public transport closures.

1.3 Apathetic care

This theme was coded eight times in Ghanaian interviews, eight times in Rwandan interviews and 13 times in South African interviews. In Ghanaian and South African interviews, reported apathetic care skewed towards the urban sample, whereas in Rwandan interviews apathetic care skewed marginally towards rurality.

Some respondents from Ghana noted they remained unattended to by healthcare professionals for hours after arriving at the final health facility and after the initial presentation. Moreover, those needing higher level care often received no care or human interaction from healthcare professionals prior to inter-facility transfer. Respondents from Ghana also noted that doctors often delegated all patient interaction to the nursing staff. Both Ghanaian and Rwandan respondents noted that nursing staff were generally inattentive to patients in pain, whilst respondents in South Africa stated nursing staff were unresponsive to patient needs throughout the nightshift. Respondents in Rwanda and South Africa stated that healthcare professionals could sometimes become quarrelsome or aggressive when patients made enquiries regarding their care.

1.4 Paired theme: individual financial scarcity & inadequate health insurance coverage

These themes were present 19 times in Ghanaian interviews, 17 times in Rwandan interviews and six times in South African interviews.

Participants struggled to finance transportation to hospitals (including ambulance services), medical care, pharmaceutical costs, and follow-up expenses. Respondents in Rwanda noted the selling of wealth (such as livestock and arable land) to fund medical treatment, whilst respondents from Ghana noted foregoing medical care due to financial barriers. In South Africa, unsuccessful disability and/or unemployment benefit applications resulted in financial barriers to follow-up care for some. Inadequate healthcare coverage affected numerous Ghanaian respondents who reported to be either uninsured or holding lapsed health insurance. Some Rwandan respondents reported that the tiered nature of their health insurance system provided inadequate coverage of certain healthcare services (e.g. one polytrauma patient received management for their musculoskeletal injury but not their dental trauma).

For peer review only - http://bmjopen.bmj.com/site/about/guidelines.xhtml

1.5 Aggregated theme: Health literacy & information giving

Health literacy (as compared against authors' knowledge of health services and understood as personal characteristics and social resources needed to access, understand and use healthcare information and services) was coded 10 times in Ghanaian interviews, three times in Rwandan interviews and was not coded in South African interviews. Inadequate information giving was coded 10 times in Ghanaian interviews, five times in Rwandan interviews, and 12 times in South African interviews. These themes were aggregated after coding.

Some Ghanaian respondents reported that injured patients were taken to their family homes to deliberate on next steps, rather than directly to definitive care. Health centres were often used as the first port-of-call after injury as opposed to hospitals due to their relative proximity. They reported that hospital care was reserved for those with head injuries, loss of consciousness, substantial bleeding, or soft tissue injury. Other Ghanaian interviewees reported that hospitals were inappropriate for fracture management and medical intervention may lead to limb deformity or amputation. These individuals perceived that injury patients would be spared from these complications by using local, bone-setting services.

The Ghanaian respondents also stated that clinicians did not provide them with any information regarding their diagnosis, management plan or expected fit-to-work date. Some Rwandan respondents noted no explanations were provided for their early discharge, their follow-up plan or why they had no follow-up appointments scheduled. South African respondents reported that there was no information given on how to access inter-hospital transportation or their self-care requirements. One South African respondent noted no explanation was offered regarding their surgical error.

2. Common facilitators

Six broad facilitators were shared and prevalent across at least two countries (Table 2). Less prevalent facilitators are only reported with their thematic frequencies in appendices (append.7-9).

2.1 Information giving and informed consent

These themes were paired prior to coding and were coded four times in Ghanaian interviews, 12 times in Rwandan interviews and eight times in South African interviews.

For peer review only - http://bmjopen.bmj.com/site/about/guidelines.xhtml

Ghanaian and South African respondents noted that clinical staff informed them about their diagnosis and imaging results. Their proposed management plans, including the regularity of bandage redressing, medications indicated and required surgical procedures were also explained. Respondents from Rwanda, particularly those in an urban setting, commented on the time given to patients to deliberate on treatment decisions, together with clinicians' support of patients' refusal of treatment after initial consent. Respect of patients' preference in the decision-making process with periodic clarification of understanding was also noted amongst Rwandan respondents.

2.2 Health insurance

This theme was coded four times in Ghana, 17 times in Rwanda and once in South Africa.

The utilisation of health insurance in Rwanda was common in interviews, whereas its utilisation in South Africa was reported once. Across all countries health insurance was noted to increase accessibility to healthcare services as well as the range of healthcare services available. Health insurance enabled extended in-patient stay, whilst making out-of-pocket payments manageable. In Rwanda, certain types of insurance were considered more beneficial than others, including "*Community-Based Health Insurance*" and *'RAMA'* (a civil service insurance scheme). Charity also played a role in insurance provision in rural Rwanda, most notably *Partners in Health,* which was active in the Burera area when the rural study was conducted.

2. 3 Health literacy

This theme was coded three times in Ghanaian interviews, nine times in Rwandan interviews and was not coded in South African interviews.

Rwandan respondents (in contrast to Ghanaian respondents) primarily utilised hospital over bone-setting services after acute injury, implying that this was the norm in Rwanda. Respondents noted that in cases where an individual bystander suggested traditional methods, they tended to be overruled by group consensus. One Rwandan interviewee noted that traditional healing may lead to long-term complications, which would necessitate medical management. A Rwandan respondent suggested that ambulance care was ideal as ambulances were given priority access in traffic, whilst another eluded to the benefit of initial patient management in an ambulance.

2.4 Paired theme: Empathetic & responsive care

These themes were paired prior to coding and were coded twice in Ghanaian interviews, 10 times in

For peer review only - http://bmjopen.bmj.com/site/about/guidelines.xhtml

Rwandan interviews and eight times in South African interviews. One Ghanaian respondent noted that healthcare professionals displayed a gentle manner, explored patients' feelings and showed concern for their work, whilst another noted that doctors tried to inspire their patients. Rwandan respondents noted that healthcare professionals were responsive to their needs. This included providing analgesia in response to pain, allowing a carer to stay at a patient's bedside overnight and addressing and alleviating a patient's concern about the possibility of amputation. One Rwandan respondent commented on the provision of a counsellor who would converse with patients and assess their wellbeing. Respondents stated that healthcare professionals did not complain of tiredness, spoke with patients throughout procedures, with one healthcare professional accompanying a patient on their hospital transfer.

South African respondents in a rural setting noted that healthcare professionals checked patients' condition regularly, communicated in an understanding manner, provided bedpans for those who struggled to reach the toilet and regularly changed sheets in response to bleeding.

2.5 Aggregated theme: Multidisciplinary management & discharge planning

Multidisciplinary management was coded twice in Ghanaian interviews, twice in Rwandan interviews and 10 times in South African interviews. Discharge planning was coded once in Ghanaian interviews, five times in Rwandan interviews and nine times in South African interviews.

One Ghanaian respondent noted that physiotherapists provided them with a *'Zimmer'* frame for physical rehabilitation, whilst a Rwandan respondent noted that a physiotherapist attended their home and built equipment to practise mobilising. Physical therapy featured in many South African interviews, with activities including resistance band training and home exercises. This involved both in-patient and out-patient therapy, often on a regular basis. One South African respondent noted they received occupational therapy input too.

Rwandan respondents in an urban setting and South African respondents in both settings mentioned discharge planning. The Rwandan respondents noted that planned follow-up appointments were arranged for patients prior to discharge. Successive appointments enabled doctors to monitor progress (assisted by imaging) and address issues through prescribing and nurse-led wound redressing. South African respondents received clinician follow-up as well as specific dates for stitch/cast removal, wound redressing, and physiotherapy.

For peer review only - http://bmjopen.bmj.com/site/about/guidelines.xhtml

2.6 Aggregated theme: Informal transportation & ambulance transportation

These two themes were aggregated after coding. Informal transportation was coded 14 times in Ghanaian interviews, 12 times in Rwandan interviews and 16 times in South African interviews. Ambulance transportation was coded three times in Ghanaian interviews, 14 times in Rwandan interviews and seven times in South African interviews.

Respondents from all three countries relied heavily on passing-by motorcycles, tricycles, and cars to reach definitive care. Reasons for seeking this form of transportation included its ready availability, its timeliness to arrival at (and its proximity to) the site of injury, its relative affordability, and local norms. Ghanaian respondents noted that injured patients' family members often used their own vehicles, borrowed vehicles, or sought known drivers in the region to transport their relative to emergency care. Transportation of injured Rwandan respondents was sometimes facilitated by the police, the army, or by a doctor. In rural Rwandan settings that were inaccessible to vehicles, transportation was afforded using traditional stretchers known as "ingobyi”. Some South African respondents reported driving themselves to acute care or being transported by their employer. Informal transportation was also employed for inter-hospital transfers and reaching follow-up appointments. South African respondents also noted utilising friends, family, and neighbours to reach follow-up care, whilst participants across the three countries utilised public transportation (including bus and taxis) to reach follow-up care.

3. Discordant data

Two barriers and one facilitator were identified that were prevalent in one country only. The barrier, ‘*cultural value of alternative services*’ was coded 12 times in Ghanaian interviews - seven times in rural settings and five times in urban settings. The barrier ‘*bystander effect’* was coded six times in Rwandan interviews, evenly distributed between rural and urban settings. The facilitator *‘shared ownership of management plan’* was coded five times in rural South African interviews.

4. Rural versus urban findings

Across all three countries, the most prevalent barriers in urban settings tended to be mirrored in rural settings *Service and treatment availability* was coded 20 times in urban settings and 19 times in rural settings, *individual financial scarcity and inadequate health insurance coverage* was coded 20 times in urban settings and 22 times in rural settings, and *inadequate information giving* was coded 13 times in urban settings and 14 times in rural settings. Two prevalent barriers were shared amongst urban and rural settings but more frequent in urban areas: *transportation barriers* were coded 25 times in

For peer review only - http://bmjopen.bmj.com/site/about/guidelines.xhtml

urban settings and 18 times in rural settings, whilst *apathetic care* was coded 18 times in urban settings and 11 times in rural settings.

Across all three countries, the most prevalent facilitators in urban settings were also prevalent in rural settings. *Informal transportation* was coded 20 times in urban settings and 22 times in rural settings, *Ambulance transportation* was coded 10 times in urban settings and 14 times in rural settings, *Health insurance* was coded nine times in urban settings and 13 times in rural settings, *Empathetic & Responsive Care* was coded nine times in urban settings and 11 times in rural settings, and *Discharge planning* was coded 9 times in urban settings and six times in rural settings. One prevalent facilitator skewed towards urbanity whilst another skewed towards rurality: *Information giving and informed consent* was coded 16 times in urban settings and eight times in rural settings, whilst *financial leniency or charity* was coded twice in urban settings and nine times in rural settings. This latter theme involved examples of ambulances not charging for transportation, clinicians not charging acute injury care and physiotherapists not charging outpatient care for individuals with limited financial resources in Ghana and Rwanda.

Discussion

We identified five common barriers and six common facilitators affecting patient experiences of accessing and receiving high-quality injury care in Ghana, Rwanda and South Africa. The barriers encompassed issues such as limited-service availability, transportation challenges, indifferent caregiving, financial constraints, and inadequate health insurance coverage, alongside low health literacy and information provision. Conversely, facilitators included effective information dissemination and informed consent practices, access to health insurance, improved health literacy, compassionate and responsive care, comprehensive multidisciplinary management and discharge planning, as well as both informal and formal transportation options including ambulance services. We discuss our findings below with reference to wider literature on seeking and accessing care in LMICs.

Individual financial scarcity and the associated theme of inadequate health insurance coverage were predominant barriers across all three countries. These barriers likely contributed to poorer patient experiences of injury care by erecting barriers to seeking, reaching, receiving, and remaining in healthcare [14]. They notably led to a lack of financial risk protection, which can increase the likelihood of injured patients forgoing care or opting for traditional care [15].

For peer review only

For peer review only - http://bmjopen.bmj.com/site/about/guidelines.xhtml

Interestingly, the response of injured patients to a lack of financial risk protection appeared to vary between countries. Amongst Ghanaian respondents, those without the capacity to pay tended to forgo care or incur costs. out o. Forgone care may have led to many injured Ghanaians acquiring disability amenable to healthcare – that is, potentially preventable given timely, effective injury care [4, 16]. Their disability severity in turn may have affected their capacity to work and therefore finance future healthcare. In this vein, Mock et al. noted that 72% of injured Ghanaian patients reported a loss of income, primarily affected by the length of ensuant disability [17].

Similarly, while some Rwandan respondents also reported forgoing care, others resorted to extreme measures such as selling or borrowing against their assets (e.g., livestock and arable land) to cover acute injury expenses, thereby jeopardizing their ability to afford future healthcare interventions and potentially exacerbating disability outcomes. The situation is compounded by the limitations of community health insurance, which, despite providing coverage for certain medical expenses, fails to address additional financial burdens such as transportation costs, caregiving expenses, and lost wages, as observed by Niyigena et al. [18]. Sapkota et al. have previously described a vicious cycle of chronic disease and poverty in Nepal. They argued convincingly that in LMICs with significant out-of-pocket expenditure for healthcare, chronic disease and poverty become mutually reinforcing across the life course [19].

Although South African respondents in our study did not report issues of insurance inadequacy or foregoing acute care due to financial constraints, financial barriers to follow-up care were noted, suggesting that while health insurance coverage may be more prevalent in this context, challenges in accessing comprehensive care persist, particularly in rural areas. In this respect, it becomes imperative to address the systemic disparities that hinder equitable access to comprehensive healthcare, especially in underserved rural regions, where the lingering financial barriers impede timely access to care and follow-up.

Availability of transportation emerged as a common theme across the study countries. While formal ambulance systems did not enable all injury patients to reach definitive care, informal methods offered some support. Particularly in Ghana, the absence of adequate ambulance services was felt most strongly amongst the respondents. However, it's worth noting that the reported access to ambulance services does not seem to translate into service uptake (at least in our small sample). Interestingly, research conducted in South Africa suggests that merely increasing the number of ambulances may not substantially improve response times, echoing sentiments from urban Cape

For peer review only - http://bmjopen.bmj.com/site/about/guidelines.xhtml

Town respondents in our study [20]. They suggested that ambulance coverage adequacy might hinge more on the perceived safety of ambulance personnel than on their sheer availability.

In contexts like rural Rwanda, where mountainous terrain renders traditional ambulances impractical, the development of community-based transportation systems, such as utilizing traditional stretchers, could be more viable. However, it's essential to ensure that such initiatives are not merely tokenistic gestures but involve genuine community participation in health system reforms, as observed in some instances in sub-Saharan Africa. Addressing these issues is crucial for supporting injury transportation systems effectively and ensuring equitable access to care across diverse geographies [21]. Strengthening community engagement and tailoring interventions to local needs could enhance the effectiveness of transportation solutions in sub-Saharan Africa.

Once at the healthcare facility, myriad barriers pertaining to service and treatment availability were highlighted across all three countries. Respondents noted the absence of care giving and unavailability of essential services such as transfusion and imaging services. These issues were often further compounded by medicinal and dressing shortages. The absence of essential medicines in hospitals and affiliated pharmacies can contribute to increased out-of-pocket expenditure of injury patients (for both pharmaceuticals and the travel costs to dispensaries).. Research suggests that '*stockouts'* affect patient safety through increasing patient exposure to substandard and falsified (SF) medicines, which are estimated to form 13.6% of drug usage in LMICs [22]. They also lead to hospital underutilisation, poorer patient experiences of care, and distrust of healthcare professionals [23]. Whilst imaging and medication may ultimately be available in some hospitals and pharmacies respectively, they may not be affordable, hence their access will be less timely, affecting patient experiences and outcomes. In this respect, the availability of services and medicines and the affordability of episodes of healthcare lie on a continuum of access barriers to quality injury care.

In all three countries, respondents highlighted concerns regarding apathetic care, while instances of compassionate care were praised. The interactions with healthcare professionals profoundly shapes patients' perceptions of quality of care, with research indicating that positive patient experiences are influenced by the disposition of doctors, their tone of voice, their attitudes towards patients and their attentiveness [24]. Cultural awareness is also important in injury care settings, given the diverse patient population encountered, particularly in a country like Ghana with significant internal migration. This migration, affecting 40% of the population, results in rural-to-urban migrants facing linguistic, cultural, and financial challenges. Acknowledging the diversity of ways of knowing

For peer review only - http://bmjopen.bmj.com/site/about/guidelines.xhtml

(epistemologies) across different Global South contexts is crucial, particularly regarding the preference for traditional bone-setting services in certain sub-Saharan African populations, rooted in historical practices [25]. Hence, any effort to enhance trauma systems should not marginalize traditional knowledge but rather integrate it into the broader healthcare framework. It is worth emphasising that treatment pathways of individual injury patients are complex and often involve multiple transitions between traditional and medical services [26]. It may be more appropriate therefore to consider these services as two components of a single health system with opportunities for complementary work, rather than discrete competing treatment decisions. For instance, recent research indicates that traditional bone setters are receptive to engagement with orthopaedic services, offering opportunities to mitigate complications through joint management approaches [27]. One potential avenue for enhancing access to quality injury care could involve integrating rehabilitative physiotherapy services into bone-setting centres, fostering synergistic partnerships for improved patient outcomes.

We note that some barriers were also framed as facilitators even, sometimes, by the same participants in interviews suggesting these are different sides of the same coin. This was especially the case for transportation, where informal transportation, when available is a facilitator, whereas when unavailable is seen as a barrier. Likewise for apathetic and empathetic care. Although many of these similar themes were reported more frequently as barriers than facilitators, these findings suggests that with deeper understanding of the context, it may be possible to turn the barriers into facilitators. For example, by formalising informal transport or by holding up examples of empathetic care and the benefits of this for others to learn from.

Finally, our analysis shows that when examining patient experiences, barriers and facilitators to accessing injury care are comparable across diverse contexts. The findings suggest that there are universal factors influencing patient perceptions, independent of the context or healthcare system. This underscores the importance of recognizing and understanding these common barriers and facilitators to inform policy decisions and develop transferable interventions aimed at improving access to quality injury care, ultimately leading to better outcomes and quality of care throughout sub-Saharan Africa. However, such interventions require to be co-designed by and with patients [28]. By integrating patients' perspectives and experiences, interventions can be customized to meet their specific needs and preferences, leading to improved clinical outcomes and a reduction in healthcare disparities [29, 30].

For peer review only - http://bmjopen.bmj.com/site/about/guidelines.xhtml

Limitations & strengths

The data collection for this study was conducted during periods of national COVID-19 lockdown, which may have transiently affected injury epidemiology. During their lockdown, South Africa introduced an alcohol ban, which limited exposure to a significant risk factor for injury [31]. The pandemic also precluded respondent validation of transcribed interviews. Moreover, we did not stratify our analyses by gender due to the small sample size of women recruited, which would have risked deanonymizing participants. These limitations have gender equity implications; however, we recognise also that most injuries are suffered by men, which is reflected by their dominance in our sample [1]. Whilst rural-urban divisions were explored in our study, large areas of each country were left unsampled, limiting national representativeness. The absence of ethnic coding of transcripts prohibited any investigation of ethnic inequities in injury care, which are known to exist for myriad health conditions. Further research on patients' experiences of injury care employing an *intersectional lens,* segmenting data by gender, ethnicity, and socioeconomic status, may illuminate any undocumented inequities.

A strength of our study was that its design was informed by prior stakeholder engagement in Ghana, Rwanda, and South Africa [8, 32]. Community leaders were also consulted prior to the study and involved in various aspects of the research. The credibility of our study has been improved through using multiple analysts and through triangulating our findings with the published literature. Investigator checking was undertaken at multiple stages throughout the thematic coding process. Our prior mixed-methods study in Ghana, Rwanda and South Africa has been used for methodological and data source triangulation. 20/34 barriers (58.8%) in our study and 13/25 facilitators (52%) were identified in this prior study, (append. 10) strengthening the confidence in the validity of these findings [8].

Conclusion

Our analysis of patient experiences across Ghana, Rwanda, and South Africa revealed consistent themes of both barriers and facilitators in accessing and receiving high-quality injury care. Financial scarcity and inadequate health insurance coverage, transportation accessibility, service and treatment availability emerged as predominant barriers across all three countries, contributing to poorer patient experiences. and potentially leading to a lack of financial risk protection. Facilitators included effective information giving and informed consent practices, access to health insurance, improved health literacy, empathetic and responsive care, comprehensive multidisciplinary management and discharge planning, as well as both informal and formal transportation options including ambulance

For peer review only

services. Our findings suggest that there may be universal factors influencing patient perceptions of quality care, regardless of the country or healthcare system, and highlight the importance of understanding common barriers and facilitators to inform policymaking and develop effective transferable strategies for improving patient experiences of injury care in sub-Saharan African countries.

Acknowledgements

We would like to acknowledge all participants in our study, who generously shared their time, experiences and insights with us.

Authors

Equi-Trauma Collaborative:

Ciaran Kennedy[1], Agnieszka Ignatowicz[1], Maria Lisa Odland[1,2,3,4,5], Abdul-Malik Abdul-Latif[1,6], Antonio Belli[7,8], Anthony Howard[9,10], John Whitaker[1,11], Kathryn M. Chu[12,13], Karen Ferreira[12], Eyitayo O. Owolabi[12], Samukelisiwe Nyamathe[12], Stephen Tabiri[14,15,16], Bernard Ofori[14], Sheba Mary Pognaa Kunfah[14], Mustapha Yakubu[15,17], Abebe Bekele[18,19], Barnabas Alyande[20,21], Pascal Nzasabimana[22], Jean-Claude Byiringiro[22,23] and Justine Davies[1,12,24]

1. Institute of Applied Health Research, University of Birmingham, Birmingham, UK

2. Department of Obstetrics and Gynaecology, St. Olavs University Hospital, Trondheim, Norway

3. Malawi-Liverpool-Wellcome Trust Research Institute, Blantyre, Malawi

4. Department of Public Health and Nursing, NTNU, Norwegian University of Science and Technology, Trondheim, Norway

5. Institute of Life Course and Medical Sciences, University of Liverpool, Liverpool, United Kingdom

6. Volta Regional Health Directorate, Ghana Health Service, Accra, Greater Accra, Ghana

7. Institute of Inflammation and Ageing, University of Birmingham, Birmingham, UK

8. National Institute for Health Research Surgical Reconstruction and Microbiology Research Centre, Birmingham, UK

9. Leeds Institute of Rheumatic and Musculoskeletal Medicine, School of Medicine, University of Leeds, Leeds, UK

10. Nuffield Department of Orthopaedics, Rheumatology and Musculoskeletal Sciences, National Institute of Health Research (NIHR) Biomedical Centre, University of Oxford, Headington, Oxford, UK

11. King's Centre for Global Health and Health Partnerships, King's College London Faculty of Life Sciences and Medicine, London, UK

For peer review only - http://bmjopen.bmj.com/site/about/guidelines.xhtml

For peer review only

12. Centre for Global Surgery, Department of Global Health, Stellenbosch University, Cape Town, South Africa

13. Department of Surgery, University of Botswana, Gaborone, Botswana

14. Ghana HUB of NIHR Global Surgery, Tamale, Ghana

15. Department of Public Health, Tamale Teaching Hospital, Tamale, Ghana

16. Department of Surgery, Tamale Teaching Hospital, Tamale, Ghana

17. School of Medicine and Health Sciences, University for Development Studies, Tamale, Ghana

18. University of Global Health Equity, Kigali, Rwanda

19. Department of Surgery, Addis Ababa University, Addis Ababa, Ethiopia

20. Center for Equity in Global Surgery, University of Global Health Equity, Kigali, Rwanda

21. Program in Global Surgery and Social Change, Harvard Medical School, Boston, Massachusetts, USA

22. University of Rwanda, Kigali, Rwanda

23. Department of Surgery, University Teaching Hospital of Kigali, Kigali, Rwanda

24. Medical Research Council/Wits University Rural Public Health and Health Transitions Research Unit, Faculty of Health Sciences, School of Public Health, University of the Witwatersrand, Johannesburg, South Africa

Contributors

CK conducted the analyses and wrote the initial draft. JD and AI developed the idea for this analysis and supervised CK in the analyses and writing. JD, KC, ST and JCB developed the protocol for the main study and oversaw it. JW, ABell, AH contributed ideas for the protocol and running of the project. MLO, AI, A-MA-L, AB, BA, JCB, ST, KC and JD oversaw the day-to-day data collection and supervised the data collectors. KF, EOO, SN, BO, SMPK, MY and PN collected data for the study. All authors have reviewed and approved the manuscript. JD is the guarantor.

Funding

This research was funded by the National Institute for Health and Care Research grant number 130036.

Competing interests

None declared.

Data availability statement

For peer review only

For peer review only - http://bmjopen.bmj.com/site/about/guidelines.xhtml

Data are available upon a reasonable request from Prof Justine Davies (email: j.davies.6@bham.ac.uk).

Patient consent for publication

Verbal and signed consent obtained directly from all participants.

Ethics approval

The overall study was approved by University of Birmingham Research Ethics Committee, UK (ERN_20-00880). Data collection in each individual country were approved by appropriate Ethics Review Boards: Ghana Health Service Ethics Review Committee (GHS-ERC005/02/20); The Stellenbosch University Health Research Ethics Committee for South Africa (Reference: N20/01/010) and the IRB of the College of Medicine and Health Sciences of the University of Rwanda for Rwanda (No 050/CMHS IRB/2020). Additional approval was obtained from the Western Cape Department of Health (Reference: WC_202006_022) in South Africa, and in the other countries approval was sought from the respective hospitals before visiting the facilities.

Provenance and peer review

Not commissioned; externally peer reviewed.

References

1. WHO, *Injuries and violence factsheet.* 2021.
2. Ryan-Coker, M.F.D., et al., *Economic burden of road traffic injuries in sub-Saharan Africa: a systematic review of existing literature.* BMJ Open, 2021. **11**(9): p. e048231.
3. Mock, C., et al., *An estimate of the number of lives that could be saved through improvements in trauma care globally.* World J Surg, 2012. **36**(5): p. 959-963.
4. Edem, I.J., et al., *External injuries, trauma and avoidable deaths in Agincourt, South Africa: a retrospective observational and qualitative study.* BMJ Open, 2019. **9**(6): p. e027576.
5. Hollis, S.M., et al., *Tracking global development assistance for trauma care: A call for advocacy and action.* J Glob Health, 2021. **11**: p. 04007.
6. WHO, *SDG Target 3.6: Road Traffic Injuries*.
7. Whitaker, J., et al., *Assessing trauma care systems in low-income and middle-income countries: a systematic review and evidence synthesis mapping the Three Delays framework to injury health system assessments.* BMJ Glob Health, 2021. **6**(5).
8. Collaborative, T.E.-T., et al., *Equitable access to quality trauma systems in low-income and middle-income countries: assessing gaps and developing priorities in Ghana, Rwanda and South Africa.* BMJ Global Health, 2022. **7**(4): p. e008256.
9. Whitaker, J., et al., *Assessing barriers to quality trauma care in low and middle-income countries: A Delphi study.* Injury, 2020. **51**(2): p. 278-285.

For peer review only - http://bmjopen.bmj.com/site/about/guidelines.xhtml

10. Tong, A., P. Sainsbury, and J. Craig, *Consolidated criteria for reporting qualitative research (COREQ): a 32-item checklist for interviews and focus groups.* International Journal for Quality in Health Care, 2007. **19**(6): p. 349-357.
11. QSR International Pty Ltd, *NVivo*. 2020.
12. Nzasabimana, P., et al., *Barriers to equitable access to quality trauma care in Rwanda: a qualitative study.* BMJ Open, 2023. **13**(9): p. e075117.
13. Owolabi, E.O., et al., *Social Determinants of Seeking and Reaching Injury Care in South Africa: A Community-Based Qualitative Study.* Ann Glob Health, 2023. **89**(1): p. 5.
14. Stanford-Moore, G.B., et al., *Effect of Delay of Care for Patients with Craniomaxillofacial Trauma in Rwanda.* OTO Open, 2022. **6**(2): p. 2473974x221096032.
15. Rahman, T., D. Gasbarro, and K. Alam, *Financial risk protection from out-of-pocket health spending in low- and middle-income countries: a scoping review of the literature.* Health Research Policy and Systems, 2022. **20**(1): p. 83.
16. Nolte, E. and M. McKee, *Measuring the health of nations: analysis of mortality amenable to health care.* Bmj, 2003. **327**(7424): p. 1129.
17. Mock, C.N., et al., *Economic consequences of injury and resulting family coping strategies in Ghana.* Accid Anal Prev, 2003. **35**(1): p. 81-90.
18. Niyigena, A., et al., *The true costs of cesarean delivery for patients in rural Rwanda: Accounting for post-discharge expenses in estimated health expenditures.* Int J Equity Health, 2022. **21**(1): p. 62.
19. Sapkota, T., I. Houkes, and H. Bosma, *Vicious cycle of chronic disease and poverty: a qualitative study in present day Nepal.* Int Health, 2021. **13**(1): p. 30-38.
20. Stein, C., L. Wallis, and O. Adetunji, *Meeting national response time targets for priority 1 incidents in an urban emergency medical services system in South Africa: More ambulances won't help.* S Afr Med J, 2015. **105**(10): p. 840-4.
21. Ankomah, S.E., et al., *Patient-public engagement strategies for health system improvement in sub-Saharan Africa: a systematic scoping review.* BMC Health Services Research, 2021. **21**(1): p. 1047.
22. Ozawa, S., et al., *Prevalence and Estimated Economic Burden of Substandard and Falsified Medicines in Low- and Middle-Income Countries: A Systematic Review and Meta-analysis.* JAMA Network Open, 2018. **1**(4): p. e181662-e181662.
23. Olaniran, A., et al., *Stock-outs of essential medicines among community health workers (CHWs) in low- and middle-income countries (LMICs): a systematic literature review of the extent, reasons, and consequences.* Hum Resour Health, 2022. **20**(1): p. 58.
24. ANANE, A., *DOCTOR-PATIENT COMMUNICATION IN THE CONSULTING ROOM: A STUDY OF TWO UNIVERSITY HEALTH FACILITIES IN GHANA*. 2021.
25. Abang, I.E., et al., *Reasons for Patronage of Traditional Bone Setters.* Niger J Surg, 2016. **22**(2): p. 102-106.
26. Dickson, D.C., et al., *A Qualitative Study of Transitions Between Health Care Settings After Injury in Cameroon.* Journal of Surgical Research, 2019. **244**: p. 528-539.
27. Garikapati, V., et al., *Systematic review of complications associated with treatment by traditional bone setters for musculoskeletal injury.* Trop Doct, 2023. **53**(1): p. 13-19.
28. Okeny, P.K., et al., *Dimensions of patient-centred care from the perspective of patients and healthcare workers in hospital settings in sub-Saharan Africa: A qualitative evidence synthesis.* PLoS One, 2024. **19**(4): p. e0299627.
29. Ibbotson, J.L., et al., *Overcoming Barriers to Accessing Surgery and Rehabilitation in Low and Middle-Income Countries: An Innovative Model of Patient Navigation in Nepal.* World J Surg, 2021. **45**(8): p. 2347-2356.
30. Freund, K.M., et al., *Impact of patient navigation on timely cancer care: the Patient Navigation Research Program.* J Natl Cancer Inst, 2014. **106**(6): p. dju115.

For peer review only

For peer review only - http://bmjopen.bmj.com/site/about/guidelines.xhtml

31. Chu, K.M., et al., *Trauma trends during COVID-19 alcohol prohibition at a South African regional hospital.* Drug Alcohol Rev, 2022. **41**(1): p. 13-19.

32. Odland, M.L., et al., *Identifying, Prioritizing and Visually Mapping Barriers to Injury Care in Rwanda: A Multi-disciplinary Stakeholder Exercise.* World J Surg, 2020. **44**(9): p. 2903-2918.

For peer review only

For peer review only - http://bmjopen.bmj.com/site/about/guidelines.xhtml

Appendices

Appendix 1. The English version of the topic guide

Equitable access to quality trauma systems in Lower and Middle Income Countries. Assessing gaps and developing priorities

Service User In-Depth Interview Topic Guide Version 1. 25-01-2020 (note this is a discussion guide, the research assistants running the study will be fluent in English and local languages, and will translate discussion points, where needed, for participants)

First, I would like to thank you for attending this interview.

The recordings will be anonymous, and we don't want you to say your name at the start, but we have given each of you a pseudonym (made up name), and will go around the table in turn asking you to say your pseudonym, what age you are, the area in which you live.

This interview is to understand what would happen in <u>your community</u> if someone suffered an injury.

I would like to think particularly about four different aspects of what would happen, these aspects are <u>seeking care</u> (the time from an injury happens to taking action to get care), <u>reaching care</u> (the time until a facility or hospital that can treat the injury is reached), <u>receiving quality care</u> (getting good quality treatment for the injury at a facility or hospital), and <u>remaining in care</u> (for example, attending follow up at out-patients or going to rehabilitation).

I wish to know about your experiences and also your thoughts on whether others in your community have similar experiences.

I realise that you have had an injury in the last 6 months, could you tell us about what happened to cause the injury?

Where were you when the injury happened?

How about after your injury happened, what did you do?
<u>Prompts, if needed:</u>
- Did you think that you needed help?
- Was anyone there to help?
- Were you able to telephone for help?

Did you think that you needed to go and get treatment?
(If not, why not?)

Were there any things that stopped you trying to get treatment?
<u>Prompts, if needed</u>
- Knowledge of availability of healthcare?
- Trust in healthcare?
- Fear of costs?
- Availability of a phone?
- No time to go to healthcare?

Where did you first go to for treatment?
<u>Prompts, if needed:</u>
- Is that a clinic or a hospital?
- How far away was it?
- How long did it take to get there?
- If it took a long time to reach the care, why was that?

Was that the only place you went to, or did you need to (or were you referred to) another place to get care?

For peer review only - http://bmjopen.bmj.com/site/about/guidelines.xhtml

Note to interviewer: please explore how many places the person needed to go to in order to get definitive care

Were there any things in particular that delayed you getting to a hospital or clinic for care?
Prompts, if needed:
- Ambulance availability?
- Road conditions?
- Personal safety?
- Costs?

How about the care that you got in the facilities that you attended – do you think that the medical treatment was good?
Prompts, if needed:
- Were you seen quickly?
- Did the doctors and nurses seem competent?
- Do you think that they had all that they needed to treat your injury well?

How about how they treated you?
Prompts, if needed:
- Were you treated with respect?
- Did the staff explain things to you?

How about costs of care?
Prompts, if needed:
- Did you have to pay for care?
- Did you have to borrow or sell anything to pay for care?

Were there any things in particular that stopped you getting the right treatment in the hospital?
Prompts if needed:
- Family commitments meant had to shorten stay?
- Costs of care?
- Too far for relatives to travel to visit?

After your injury was treated, were you asked to go back to see healthcare workers about your injury? For example, doctors in a clinical appointment, or physiotherapists for rehabilitation?

If so, how far did you have to travel to get to these appointments?

Did you manage to keep all of the appointments?

Were there any things in particular that you felt limited your ability to keep all these appointments?
Prompts, if needed:
- Travel time?
- Costs?
- Other commitments?
- Thought there was no need?

Are you now back to your full fitness, do you think?

Do you think anything could have been done better?

Now that's the end of this discussion, thank-you for telling us about your experiences.
Now, we would be grateful if we could ask you some other questions about your time in the hospital or clinic. It will take another ten minutes.

For peer review only

Note to interviewer ask I-PAHC and/or O-PAHC questionnaire depending on whether the participant was admitted to hospital or was seen in a clinic, or both.

Does you have any questions for me?
Thank-you

For peer review only

Appendix 2. Barriers to good patient experience of trauma care, in descending order of frequency.

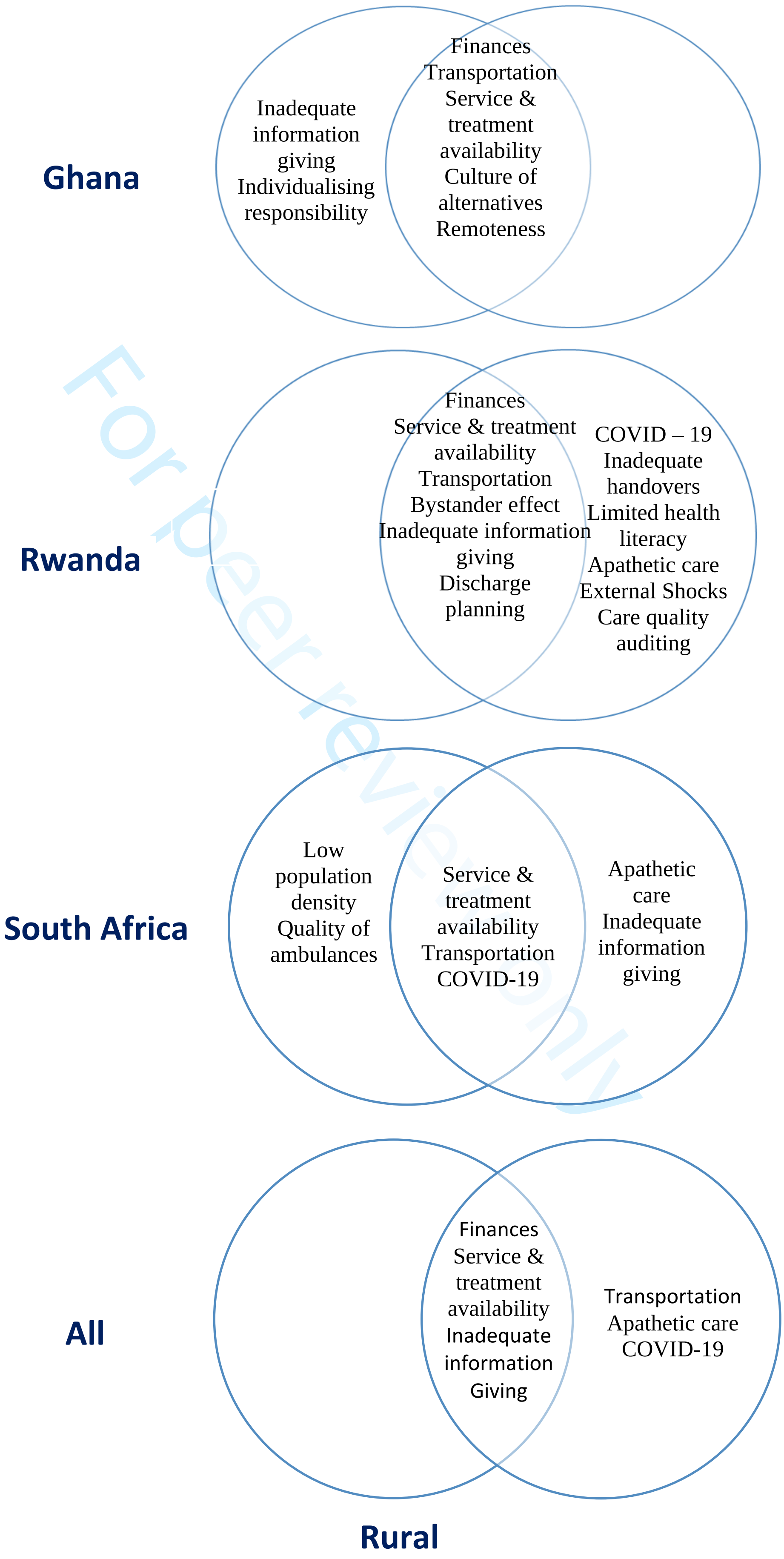

Appendix 3. Facilitators to good patient experience of trauma care, in descending order of frequency

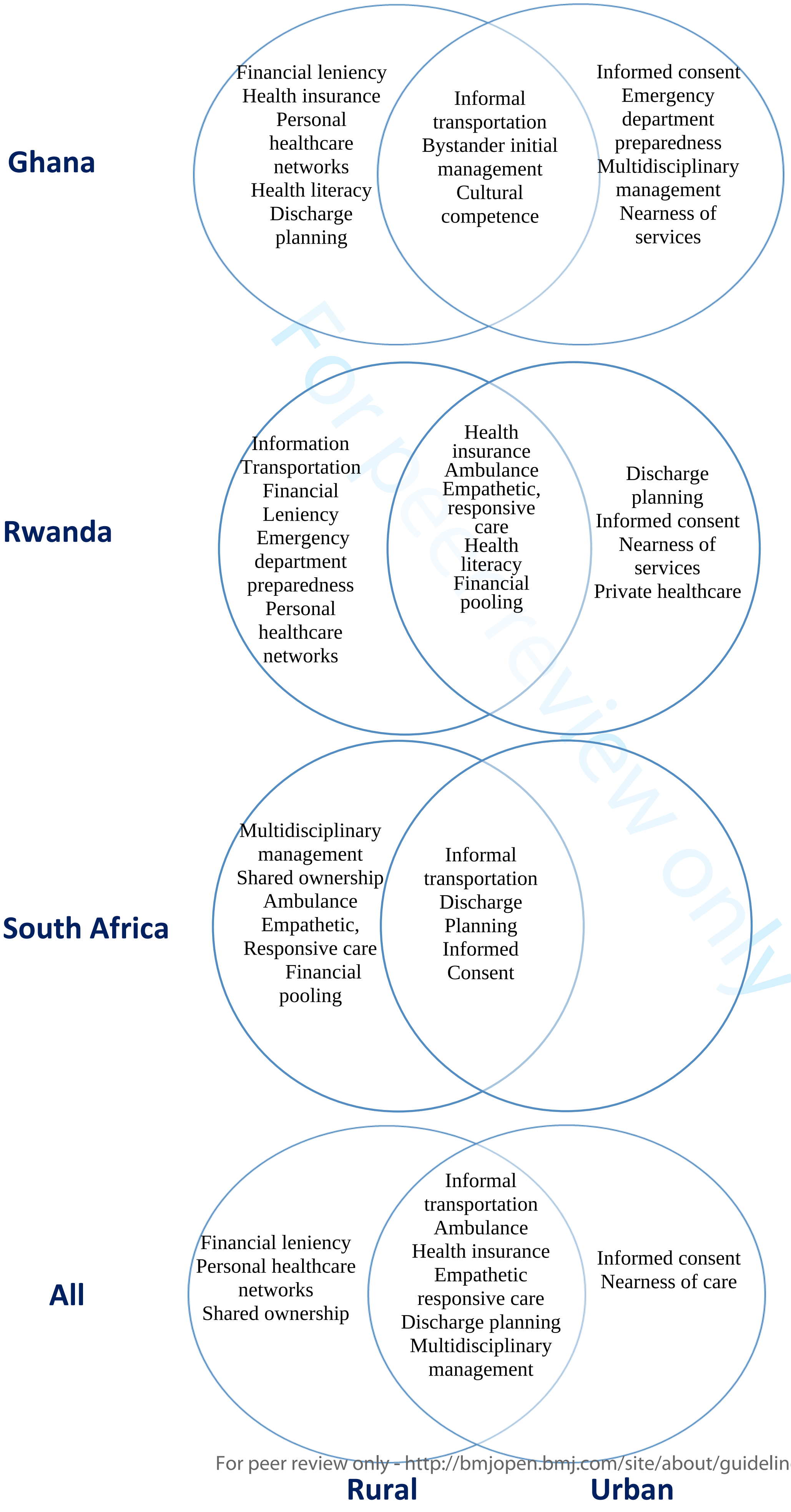

For peer review only

Appendix 4: Barriers to a good patient experience of trauma care in Ghana

	Ghana			
Barriers	Rural	Urban	Rural - Urban	Total
Financial Scarcity and inadequate healthcare coverage	10	9	1	19
Service and treatment availability	8	8	0	16
Transportation Barriers	7	8	-1	15
Cultural Value of alternative services	7	5	2	12
Inadequate Information Giving	7	3	4	10
Limited Health Literacy	2	8	-6	10
Apathetic Care	2	6	-4	8
Low Population Density	2	1	1	3
Narratives of Self-blame and Individualising Responsibility	2	0	2	2
Absence of care quality assessment	0	1	-1	1
COVID-19	0	1	-1	1
Absence of occupational health assessment	0	0	0	0
Bystander Effect	0	0	0	0
Care responsibilities	0	0	0	0
Clinician-patient power inequality	0	0	0	0
Delayed presented due to perceived low severity	0	0	0	0
Diagnostic uncertainty	0	0	0	0
Ethnic inequities in health system provision	0	0	0	0
Fear of surgery	0	0	0	0
Hospital catchment area system	0	0	0	0
Inability to self-care / loss of autonomy	0	0	0	0
Inadequate administration of Follow-Ups	0	0	0	0
Inadequate analgesia	0	0	0	0
Inadequate Discharge Planning	0	0	0	0
Inadequate Handovers	0	0	0	0
Language Barriers	0	0	0	0
Limited Specialist Training	0	0	0	0
Loss to follow up	0	0	0	0
Obstruction	0	0	0	0
Poor communication with family	0	0	0	0
Quality of ambulances	0	0	0	0
suboptimal clinical management	0	0	0	0
Unmet psychological needs	0	0	0	0
Unresponsiveness to formal complaints	0	0	0	0

For peer review only

Appendix 5: Barriers to a good patient experience of trauma care in Rwanda

	Rwanda			
Barriers	Rural	Urban	Rural - Urban	Total
Financial Scarcity and inadequate healthcare coverage	9	8	1	17
Service and treatment availability	7	9	-2	16
Transportation Barriers	7	8	-1	15
COVID-19	3	8	-5	11
Apathetic Care	5	3	2	8
Inadequate Discharge Planning	4	3	1	7
Bystander Effect	3	3	0	6
Inadequate Information Giving	3	2	1	5
Inadequate administration of Follow-Ups	2	1	1	3
Limited Health Literacy	3	0	3	3
Inadequate Handovers	0	2	-2	2
Absence of occupational health assessment	2	0	2	2
Obstruction	0	1	-1	1
Language Barriers	1	0	1	1
Limited Specialist Training	1	0	1	1
Diagnostic uncertainty	1	0	1	1
Clinician-patient power inequality	1	0	1	1
Inability to self-care / loss of autonomy	1	0	1	1
Narratives of Self-blame and Individualising Responsibility	1	0	1	1
Quality of ambulances	1	0	1	1
Inadequate analgesia	1	0	1	1
Cultural Value of alternative services	1	0	1	1
Hospital catchment area system	0	0	0	0
Ethnic inequities in health system provision	0	0	0	0
Care responsibilities	0	0	0	0
Unmet psychological needs	0	0	0	0
Fear of surgery	0	0	0	0
Loss to follow up	0	0	0	0
Unresponsiveness to formal complaints	0	0	0	0
Poor communication with family	0	0	0	0
Absence of care quality assessment	0	0	0	0
suboptimal clinical management	0	0	0	0
Delayed presented due to perceived low severity	0	0	0	0
Low Population Density	0	0	0	0

For peer review only

Appendix 6: Barriers to a good patient experience of trauma care in South Africa

	South Africa			
Barriers	Rural	Urban	Rural - Urban	Total
Apathetic Care	4	9	-5	13
Inadequate Information Giving	4	8	-4	12
Service and treatment availability	4	8	-4	12
Transportation Barriers	4	4	0	8
COVID-19	4	3	1	7
Financial Scarcity and inadequate healthcare coverage	3	3	0	6
suboptimal clinical management	1	2	-1	3
Delayed presented due to perceived low severity	2	1	1	3
Inadequate analgesia	1	2	-1	3
Unresponsiveness to formal complaints	0	2	-2	2
Poor communication with family	1	1	0	2
Absence of care quality assessment	0	2	-2	2
Inability to self-care / loss of autonomy	1	1	0	2
Quality of ambulances	2	0	2	2
Low Population Density	2	0	2	2
Inadequate Discharge Planning	0	2	-2	2
Hospital catchment area system	0	1	-1	1
Ethnic inequities in health system provision	0	1	-1	1
Care responsibilities	0	1	-1	1
Unmet psychological needs	0	1	-1	1
Fear of surgery	0	1	-1	1
Loss to follow up	0	1	-1	1
Inadequate administration of Follow-Ups	0	1	-1	1
Obstruction	0	0	0	0
Language Barriers	0	0	0	0
Limited Specialist Training	0	0	0	0
Diagnostic uncertainty	0	0	0	0
Clinician-patient power inequality	0	0	0	0
Inadequate Handovers	0	0	0	0
Absence of occupational health assessment	0	0	0	0
Narratives of Self-blame and Individualising Responsibility	0	0	0	0
Bystander Effect	0	0	0	0
Limited Health Literacy	0	0	0	0
Cultural Value of alternative services	0	0	0	0

For peer review only

Appendix 7: Facilitators to a good patient experience of trauma care in Ghana

	Ghana			
Facilitators	Rural	Urban	Rural - Urban	Total
Informal transportation	6	8	-2	14
Bystander Initial Management	2	3	-1	5
Financial Leniency / Charity	4	0	4	4
Health Insurance	4	0	4	4
Information giving & informed consent	0	4	-4	4
Personal Healthcare Networks	3	0	3	3
Health Literacy	3	0	3	3
Ambulance	2	1	1	3
Cultural Competence	0	2	-2	2
Nearness of Care	0	2	-2	2
Emergency Department Preparedness	0	2	-2	2
Multidisciplinary Management	0	2	-2	2
Community or Familial Financial Pooling	0	1	-1	1
Discharge Planning	1	0	1	1
Preferential Treatment	0	0	0	0
Police-facilitated transport	0	0	0	0
Communications Network	0	0	0	0
Familial Rehabilitative Support	0	0	0	0
Patient autonomy	0	0	0	0
NGO Support	0	0	0	0
Provision of mobility aids	0	0	0	0
Requesting Timely Care	0	0	0	0
Private Healthcare	0	0	0	0
Shared ownership of management plan	0	0	0	0
Empathetic Responsive care	0	0	0	0

For peer review only

Appendix 8: Facilitators to a good patient experience of trauma care in Rwanda

	Rwanda			
Facilitators	Rural	Urban	Rural - Urban	Total
Health Insurance	8	9	-1	17
Ambulance	6	8	-2	14
Information giving & informed consent	4	8	-4	12
Informal transportation	9	3	6	12
Empathetic Responsive care	4	6	-2	10
Health Literacy	4	5	-1	9
Community or Familial Financial Pooling	3	4	-1	7
Financial Leniency / Charity	5	2	3	7
Bystander Initial Management	2	4	-2	6
Discharge Planning	0	5	-5	5
NGO Support	2	1	1	3
Communications Network	1	1	0	2
Nearness of Care	0	2	-2	2
Private Healthcare	0	2	-2	2
Multidisciplinary Management	1	1	0	2
Preferential Treatment	1	0	1	1
Patient autonomy	0	1	-1	1
Cultural Competence	0	1	-1	1
Emergency Department Preparedness	1	0	1	1
Requesting Timely Care	1	0	1	1
Personal Healthcare Networks	1	0	1	1
Police-facilitated transport	0	0	0	0
Familial Rehabilitative Support	0	0	0	0
Provision of mobility aids	0	0	0	0
Shared ownership of management plan	0	0	0	0

For peer review only

Appendix 9: Facilitators to a good patient experience of trauma care in South Africa

	South Africa			
Facilitators	Rural	Urban	Rural - Urban	Total
Informal transportation	7	9	-2	16
Multidisciplinary Management	8	2	6	10
Discharge Planning	5	4	1	9
Empathetic Responsive care	7	1	6	8
Information giving & informed consent	4	4	0	8
Ambulance	6	1	5	7
Shared ownership of management plan	5	0	5	5
Provision of mobility aids	3	0	3	3
Community or Familial Financial Pooling	3	0	3	3
Requesting Timely Care	2	1	1	3
Private Healthcare	2	1	1	3
Familial Rehabilitative Support	1	1	0	2
Bystander Initial Management	1	1	0	2
Police-facilitated transport	1	0	1	1
Patient autonomy	1	0	1	1
Emergency Department Preparedness	1	0	1	1
Personal Healthcare Networks	1	0	1	1
Financial Leniency / Charity	1	0	1	1
Health Insurance	1	0	1	1
Preferential Treatment	0	0	0	0
Communications Network	0	0	0	0
Cultural Competence	0	0	0	0
NGO Support	0	0	0	0
Nearness of Care	0	0	0	0
Health Literacy	0	0	0	0

For peer review only

Appendix 10. ***Thematic corroboration of results with the findings of the prior mixed-methods study in Ghana, Rwanda and South Africa.***

Barriers	Barriers (Odland et al.)	Facilitators	Facilitators (Odland et al.)
Absence of care quality assessment	Present	Ambulance	Present
Absence of occupational health assessment		Bystander Initial Management	Present
Apathetic Care		Communications Network	
Bystander Effect	Present	Community or Familial Financial Pooling	
Care responsibilities		Cultural Competence	Present
Clinician-patient power inequality	Present	Discharge Planning	Present
COVID-19	Present	Emergency Department Preparedness	Present
Cultural Value of alternative services	Present	Empathetic Responsive care	Present
Delayed presented due to perceived low severity		Familial Rehabilitative Support	
Diagnostic uncertainty		Financial Leniency / Charity	
Inequities in health system provision	Present	Health Literacy	Present
Fear of surgery		Health Insurance	Present
Individual financial scarcity and inadequate health insurane coverage	Present	Informal transportation	
Hospital catchment area system	Present	Information giving and informed consent	Present
Inability to self-care / loss of autonomy		Multidisciplinary Management	Present
Inadequate administration of Follow-Ups	Present	Nearness of Care	Present
Inadequate analgesia	Present	NGO Support	
Inadequate Discharge Planning	Present	Patient autonomy	Present
Inadequate Handovers		Personal Healthcare Networks	
Inadequate Information Giving	Present	Police-facilitated transport	
Language Barriers		Preferential Treatment	
Service and treatment availability	Present	Private Healthcare	
Health literacy	Present	Provision of mobility aids	
Limited Specialist Training		Requesting Timely Care	
Low Population Density	Present	Shared ownership of management plan	Present
Narratives of Self-blame and Individualising Responsibility			Coinciding themes (%)
Obstruction			52
Poor communication with family			
Quality of ambulances	Present		
Suboptimal clinical management			
Transportation Barriers	Present		
Unmet psychological needs	Present		
Unresponsiveness to formal complaints	Present		
Loss to Follow Up			
	Coinciding themes (%)		
	60.60606061		

For peer review only

COREQ (COnsolidated criteria for REporting Qualitative research) Checklist

A checklist of items that should be included in reports of qualitative research. You must report the page number in your manuscript where you consider each of the items listed in this checklist. If you have not included this information, either revise your manuscript accordingly before submitting or note N/A.

Topic	Item No.	Guide Questions/Description	Reported on Page No.
**Domain 1: Research team and reflexivity**			
Personal characteristics			
Interviewer/facilitator	1	Which author/s conducted the interview or focus group?	
Credentials	2	What were the researcher's credentials? E.g. PhD, MD	
Occupation	3	What was their occupation at the time of the study?	
Gender	4	Was the researcher male or female?	
Experience and training	5	What experience or training did the researcher have?	
Relationship with participants			
Relationship established	6	Was a relationship established prior to study commencement?	
Participant knowledge of the interviewer	7	What did the participants know about the researcher? e.g. personal goals, reasons for doing the research	
Interviewer characteristics	8	What characteristics were reported about the inter viewer/facilitator? e.g. Bias, assumptions, reasons and interests in the research topic	
**Domain 2: Study design**			
Theoretical framework			
Methodological orientation and Theory	9	What methodological orientation was stated to underpin the study? e.g. grounded theory, discourse analysis, ethnography, phenomenology, content analysis	
Participant selection			
Sampling	10	How were participants selected? e.g. purposive, convenience, consecutive, snowball	
Method of approach	11	How were participants approached? e.g. face-to-face, telephone, mail, email	
Sample size	12	How many participants were in the study?	
Non-participation	13	How many people refused to participate or dropped out? Reasons?	
Setting			
Setting of data collection	14	Where was the data collected? e.g. home, clinic, workplace	
Presence of non-participants	15	Was anyone else present besides the participants and researchers?	
Description of sample	16	What are the important characteristics of the sample? e.g. demographic data, date	
Data collection			
Interview guide	17	Were questions, prompts, guides provided by the authors? Was it pilot tested?	
Repeat interviews	18	Were repeat inter views carried out? If yes, how many?	
Audio/visual recording	19	Did the research use audio or visual recording to collect the data?	
Field notes	20	Were field notes made during and/or after the inter view or focus group?	
Duration	21	What was the duration of the inter views or focus group?	
Data saturation	22	Was data saturation discussed?	
Transcripts returned	23	Were transcripts returned to participants for comment and/or	

For peer review only

Topic	Item No.	Guide Questions/Description	Reported on Page No.
		correction?	
**Domain 3: analysis and findings**			
Data analysis			
Number of data coders	24	How many data coders coded the data?	
Description of the coding tree	25	Did authors provide a description of the coding tree?	
Derivation of themes	26	Were themes identified in advance or derived from the data?	
Software	27	What software, if applicable, was used to manage the data?	
Participant checking	28	Did participants provide feedback on the findings?	
Reporting			
Quotations presented	29	Were participant quotations presented to illustrate the themes/findings? Was each quotation identified? e.g. participant number	
Data and findings consistent	30	Was there consistency between the data presented and the findings?	
Clarity of major themes	31	Were major themes clearly presented in the findings?	
Clarity of minor themes	32	Is there a description of diverse cases or discussion of minor themes?	

Developed from: Tong A, Sainsbury P, Craig J. Consolidated criteria for reporting qualitative research (COREQ): a 32-item checklist for interviews and focus groups. *International Journal for Quality in Health Care*. 2007. Volume 19, Number 6: pp. 349 – 357

Once you have completed this checklist, please save a copy and upload it as part of your submission. DO NOT include this checklist as part of the main manuscript document. It must be uploaded as a separate file.

For peer review only
